# Surgical managements for rhegmatogenous retinal detachment: A network meta-analysis of randomized controlled trial

**DOI:** 10.1371/journal.pone.0310859

**Published:** 2024-11-14

**Authors:** Xinyu Yan, Meng Xu, Fengjun Su

**Affiliations:** 1 Shandong Traditional Chinese Medicine University, Jinan, China; 2 Health Technology Assessment Center/Evidence-Based Social Science Research Center, School of Public Health, Lanzhou University, Lanzhou, China; 3 Department of Ophthalmology, Shandong Provincial Hospital of Traditional Chinese Medicine, Jinan, China; Hangil Eye Hospital / Catholic Kwandong University College of Medicine, REPUBLIC OF KOREA

## Abstract

**Background and objective:**

Rhegmatogenous retinal detachment (RRD) is the most common ophthalmic emergency threatening vision, with an incidence ranging from 6.3 to 17.9 per 100,000 people per year. However, optimal surgical management of RRD remains controversial. This network meta-analysis compared the efficacy and safety of different surgical options in patients with RRD.

**Methods:**

We systematically searched PubMed, Embase, Cochrane Library and Web of science for randomized controlled trials (RCT) from inception to 24^th^ September 2023. Frequentist network meta-analyses with the random-effects model was used to synthesize data. The risk of bias for the included RCTs was evaluated using the Cochrane tool for assessing risk of bias, and the certainty of evidence using the Grading of Recommendations, Assessment, Development, and Evaluation approach. And we performed the network meta-analysis utilizing R 4.1.3 software and Stata 16SE.

**Results:**

A total of 19 RCTs enrolled 2589 eyes were included. With high-to-very low certainty of evidence, compared with pneumatic retinopexy (PR), scleral buckling (SB) (odd ratio (OR) = 0.52, 95% confidence interval (CI) [0.30; 0.91]), pars plana vitrectomy (PPV) (OR = 2.35, 95% CI [1.32; 4.20]), PPV+SB (OR = 2.59, 95% CI [1.32; 5.09]) and PPV combined with phacomulsification (PCV) (OR = 7.72, 95% CI [1.07; 55.87]) were more effect in improving primary reattachment rate; for postoperative 6-month vision, SB was superior to PPV+SB (mean difference (MD) = 0.14, 95% CI [0.01; 0.27]). When compared with SB, PPV (OR = 5.27, 95% CI [3.13; 8.86]) and PPV+SB (OR = 10.12, 95% CI [4.31; 23.77]) shows a higher incidence of postoperative cataract progression. Compared to PR, the same is true for PPV (OR = 7.51, 95% CI [3.33; 16.91]) and PPV+SB (OR = 14.43, 95% CI [4.97; 41.93]).

**Conclusions:**

PR appears to be associated with a lower rate of primary reattachment rate and postoperative cataract progression. In view of the small sample sizes of the included studies and the low certainty of evidence, these findings must be interpreted with caution. A large number of high-quality trials should be conducted to verify the effects of different surgical techniques in the future.

## Introduction

Rhegmatogenous retinal detachment (RRD) is the most common form of retinal detachment, the annual incidence of RRD around the world ranges from 7.98 to 18.2 per 105 person-year [1]. RRD occurs due to retinal tear formation, allowing liquefied vitreous to enter the subretinal space, causing neurosensory retinal detachment. Without treatment, it may lead to blindness in the affected eye.

Current RRD treatments aim to seal retinal breaks, allowing retinal pigment epithelial cells to resorb subretinal fluid, and the mainstay of treatment is surgery. Previously untreatable, RRD now achieves primary surgical success rates of over 80%–90% [2] with complex cases also amenable to treatment. Since the introduction of scleral buckling (SB) in 1951, SB was the standard RRD treatment due to its minimally invasive nature [3,4]. However, with the development of microincision techniques, wide-angle microscopic systems, and ancillary tools including intraocular packing (e.g., gas and silicone oil) and perfluorocarbon fluids that help drain subretinal fluid, pars plana vitrectomy (PPV) has become the dominant method to enable precise localization and treatment of retinal tears [1,5,6]. Recent data from the Wilmer Eye Institute demonstrated a shift from SB to PPV for uncomplicated RRDs from 2008–2017 [7]. Pneumatic retinopexy (PR) is another less invasive and more economical retinal surgery. It has been widely advocated for treatment of selected RRD due to its short operation time, fast postoperative recovery, no major complications [8], no hospitalization, low cost, time savings for surgeons and so on [9]. But a review aimed at re-evaluating the advantages of PR has been reported that the reoperation rate and postoperative proliferative vitreoretinopathy (PVR) in PR is much higher than in SB or PPV, which largely offsets the cost savings and puts patients in chronic vision loss and mental stress [10]. Combination SB-PPV [11] and PPV-phacomulsification [12] surgery are also increasingly utilized, providing advantages in complex RRDs and reducing reoperation rates, respectively.

However, some studies of surgical therapies have insufficient evidence and inconsistent results. For example, Heimann et al., [13] in a prospective randomized multicenter study, evaluated the possible effects of adding an encircling element to the PPV and concluded that PRD patients who underwent PPV and an extra buckle had significantly better anatomical outcomes. However, another large randomized controlled trial (RCT) did not confirm the beneficial effect of adding an encircling element on the anatomical outcome. Conversely [14], it was associated with a trend towards a delayed visual improvement. In addition, while prior systematic reviews have summarized RRD repair methods, comparisons of multiple surgeries are lacking.

Network meta-analysis (NMA) facilitates simultaneous comparison of multiple interventions, even in the absence of direct head-to-head trials. Therefore, we will conduct an NMA of randomized controlled trials to compare the efficacy and safety of various repair techniques in patients with RRD and determine the optimal surgical approach. This NMA will synthesize the highest quality evidence addressing this critical knowledge gap.

## Materials and methods

The study protocol was prospectively registered with International Prospective Register of Systematic Reviews (PROSPERO, CRD42023436920). This NMA was conducted in accordance with the Preferred Reporting Items for Systematic Reviews and Meta-analyses extension for NMA (PRISMA-NMA) [15].

### Literature search

PubMed, Cochrane Library, Web of Science and Embase database were systematically searched from their inception to 24^th^ September 2023. We utilized the following keywords to search literatures: ("Retinal Detachment" OR "Retinal Detachments" OR "Retinal Pigment Epithelial Detachment" OR "rhegmatogenous retinal detachment") AND ("Scleral Buckling" OR "Scleral Bucklings OR Vitrectomy" OR "Vitrectomies" OR "pars plana vitrectomy" OR "pneumatic retinopexy" OR "pneumoretinopexy" OR "phacoemulsification" OR "phacoemulsifications") AND ("randomized controlled trial" OR "Clinical trial" OR "Controlled Clinical trial" OR "random" OR "blind" OR "singleblind" OR "doubleblind" OR "tripleblind" OR "trebleblind"). The full search strategies were described in S1 File. In addition, we manually searched reference lists of related studies and the record in Clinical-Trials.gov to identify some additional studies [15].

### Inclusion and exclusion criteria

Predefined eligible criteria for evidence inclusion were as follows:

#### Populations

Patients with RRD were included in our review.

#### Interventions and comparator

Both intervention and control groups were treated with surgical techniques, including pars plana vitrectomy, scleral buckling, pneumatic retinopexy, PPV combined SB and PPV-phacomulsification.

#### Outcome*s*

We used retinal reattachment rate (including primary reattachment rate and final reattachment rate) at follow-up endpoint as the primary outcomes, best-corrected visual acuity at each follow-up point and incidence of postoperative complications (including PVR, missed/new breaks, cataract progression, macular pucker, macular edema) as secondary outcomes.

#### Study design

All RCTs were reported at least one set of efficacy and/or safety outcomes in the treatment group, and full-text was available in English.

Some reasons for exclusions were: (1) studies with unreported outcome, (2) comparing the efficacy of different tamponade in treating RRD, (3) follow-up studies of included studies, (4) studies were abstracts, protocols, conference proceedings, or duplicate publications, (5) non-English language studies, (6) articles that have not been peer-reviewed.

### Study selection

Two coauthors (Y.X.Y and M.X) independently screened all titles and abstracts using Rayyan (http://rayyan.qcri.org) [16,17], a free web application that speeds up initial screening with a semi-automated process, which has high usability. Next, full-text articles of potentially eligible studies were obtained, and classified as ineligible or eligible studies by two reviewers. Any disagreements are resolved through consultation with the third reviewer (F.J.S) to achieve consensus.

### Data extraction

Two coauthors (Y.X.Y and M.X) independently extracted relevant data parameters and entered them in electric format into Microsoft Excel 2019. In the event of disagreement, a third reviewer, S.F.J, arbitrated any differences that could not be resolved by consensus. When available, the following data were collected at baseline, the basic information (the name of the first author, country, study year of publication and the number of study centers) and the characteristics of participants (number of eyes, age, sex, intervention and control, distribution of right eyes, lens status, best-corrected visual acuity (BCVA), intraocular pressure (IOP), break details (inferior breaks, extension of detached quadrant), macular status, presence of lattice degeneration, vitreous hemorrage, proliferative vitreoretinopathy (PVR) grade and the target follow-up months). Besides, the following data were extracted at the follow-up endpoint of each study if available: the primary (i.e., single surgery) and final reattachment rates, BCVA, incidence of postoperative PVR, macular pucker, macular edema, postoperative cataract progression and the number of missed/new breaks. BCVA was collected in Early Treatment for Diabetic Retinopathy Study (ETDRS) letters or LogMAR (logarithm of the minimal angle of resolution) notation as per the data available in the included studies.

### Risk-of-bias assessment

The risk of bias (ROB) of included RCTs was assessed using the Cochrane Risk of Bias tool. Two coauthors (Y.X.Y and M.X) independently performed the risk-of-bias assessment on all included RCTs. In the event of disagreement, a third reviewer, F.J.S, arbitrated any differences that could not be resolved by consensus. This tool evaluated six domains, including selective bias due to lack of randomization and allocation concealment process, performance bias due to lack of blinding of participants and staff, detection bias due to lack of blinding of outcome assessors, attrition bias due to incomplete outcome data, reporting bias due to selective outcome reporting, and other sources of bias. The standard of domain ‘performance bias’ were modified due to the impossibility of blinding during surgical treatment [18,19]. The overall risk of bias was categorized according to the following: low risk of bias when five domains were judged to be ‘low’, high risk of bias if one or more domains were judged as ‘high’; others were considered as ‘some concerns about risk of bias’. Inter-rater agreement was analyzed using Cohen’s kappa coefficient (κ), when p>0.8 indicates better agreement.

### Certainty of evidence assessment

We rated the certainty of evidence for each outcomes using the GRADE (Grading of Recommendations, Assessment, Development and Evaluation) framework, which classifies evidence as high, moderate, low, or very low certainty. The starting point for certainty in direct estimates for RCTs is high but can be downgraded based on limitations for risk of bias, imprecision, inconsistency, indirectness, and publication bias. Judgements for each factor can be ‘not serious’ (not degraded), ‘serious’ (degraded by one level), or ‘very serious’ (degraded by two levels) [20–22].

### Statistical analysis

We utilized the odd ratio (OR) for dichotomous variables with 95% confidence intervals (CIs). For continuous variables, we calculated the mean and standard deviation (SD) changes according to the Cochrane Handbook when primary study just reported measurement values that pre- and post- intervention [19,23]. (S2 Text) If the data were reported in other formats, such as standard errors, p-values, confidence intervals, interquartile ranges, or figures, we estimated the missing SDs using available data from the same or other studies included in the network meta-analysis (S3 Text) [19,24]. Finally, we use mean difference (MD) with 95% CIs to express continuous variables.

NMA was performed using frequentist random-effects model NMA in the statistical software R V.4.1.3 (*netmeta* package V.2.8.0) [19]. The node splitting was conducted to assess local inconsistency. It indicated the existence of local inconsistency when *p*<0.05. We calculated the relative ranking of operations for each outcome as their surface under the cumulative ranking (SUCRA), which interpret the mean extent of certainty of that one treatment was better/lower than another. Higher SUCRA score (i.e., close to 100%) means a higher ranking for efficacy outcome, while for safety outcomes means higher ranking for adverse events/serious adverse events (i.e., lower SUCRA correlates with better safety) [25]. For example, assuming that the SUCRA score for PPV in retinal reattachment rate is 93% compared to 80% for SB, then we believe that PPV is superior in retinal reattachment rate compared to SB. However, if the same results were presented in terms of the occurrence of postoperative macular edema, it would suggest that PPV has a higher incidence of postoperative macular edema. However, due to the uncertainty of SUCRA, interpretation of SUCRA values needs to be linked to evidence from related interventions [19,25]. Furthermore, a meta-regression was performed in order to explore the effects of seven predefined variables including year of publication, grade of PVR at baseline, lens status, severity of RRD, age, sample size, and RoB grade on each outcome. For the network meta-regression, scleral buckling was the reference [16,26]. Funnel plots and Egger’s test to evaluate publication bias for each pairwise comparison if the number of studies was no less than 10 studies [19,27]. Stata 16SE was used to create a visual network map to show the associations between interventions.

## Results

### Study selection

**Fig 1:**
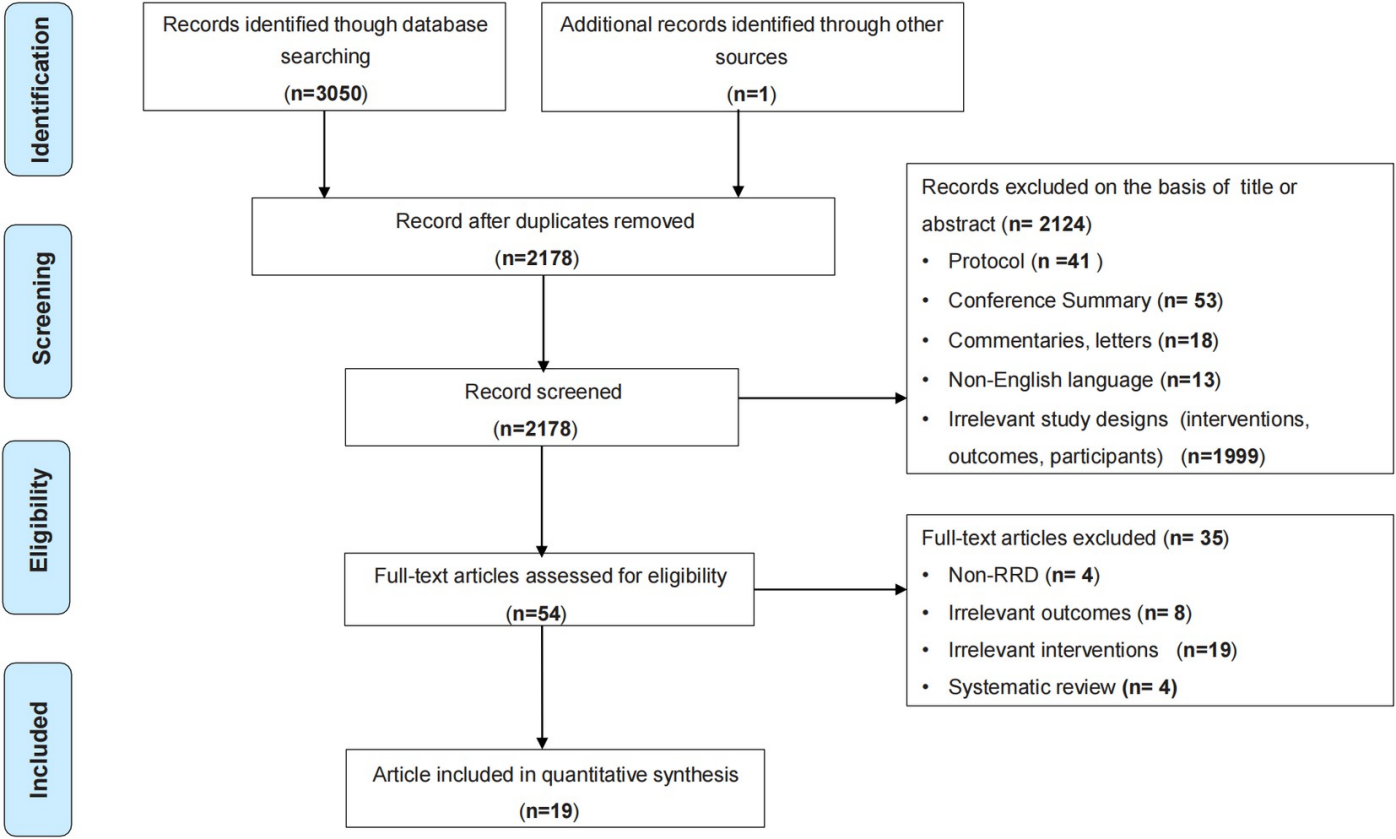
Flowchart of the Preferred Reporting Item for Meta-Analysis (PRISMA) study selection process.

A total of 3051 potential records were identified, and 2178 were screened for titles and abstracts after removing duplicates. After full-text screening of 54 potentially eligible studies 19 were included in our analysis. The literature screening process is shown in the list of eligible studies is presented in Fig 1.

### Characteristics of included studies

19 RCTs including 2589 eyes were enrolled. The average age is 50.5 years and the proportion of male patients is about 64%. Included studies were published between 1989 and 2021. Indian and Iran had the highest number of publications (both 3 RCTs) and the largest proportion of PPV (37%). Baseline logMAR BCVA ranged from 0.04 to 2.4 across the various cohorts. Overall, 5 studies included only phakic eyes, 6 included only aphakic/pseudophakic eyes, and the remaining studies were all included. 12 studies reported macular status and the proportion of macular-off eyes ranged from 45% to 98%. Studies typically excluded patients with significant PVR (17/19), uncontrolled glaucoma (8/19), uveitis (4/19), intraocular trauma (9/19), severe refractive interstitial opacity (12/19), interstitial opacity (12/19), giant retinal tears (8/19), severe diabetic retinopathy (4/19), intraocular surgery (14/19), children (7/19), and macular pathology (7/19). A complete listing of baseline demographic and clinical parameters can be found on S5 File.

### Risk-of-bias and quality-of-evidence assessment

Inter-rater agreement for RoB and certainty of evidence assessment were κ = 0.915 and κ = 0.924, respectively. The RoB assessments for the included studies is shown in S12 File. Low RoB was assessed in 13 studies (68.42%) and 7 studies (36.84%) for the process of generating the sequence meeting the requirements of randomization and allocation concealment, respectively, in all studies (100%) for reporting the predefined outcomes, blinding of participants and personnel and selective outcome reporting, and 8 studies for reporting other sources of bias. However, the item of sequence generating and allocation concealment for 1 study (5.26%) were rated as high risk of bias due to sequences generated from hospital records. Overall, there were 13 (68.4%), 5 (26.3%), and 1 (5.3%) studies that had a unclear, low, and high risk of bias, respectively. The detailed results of GRADE scoring are shown in S6 File.

### Network plots

**Fig 2:**
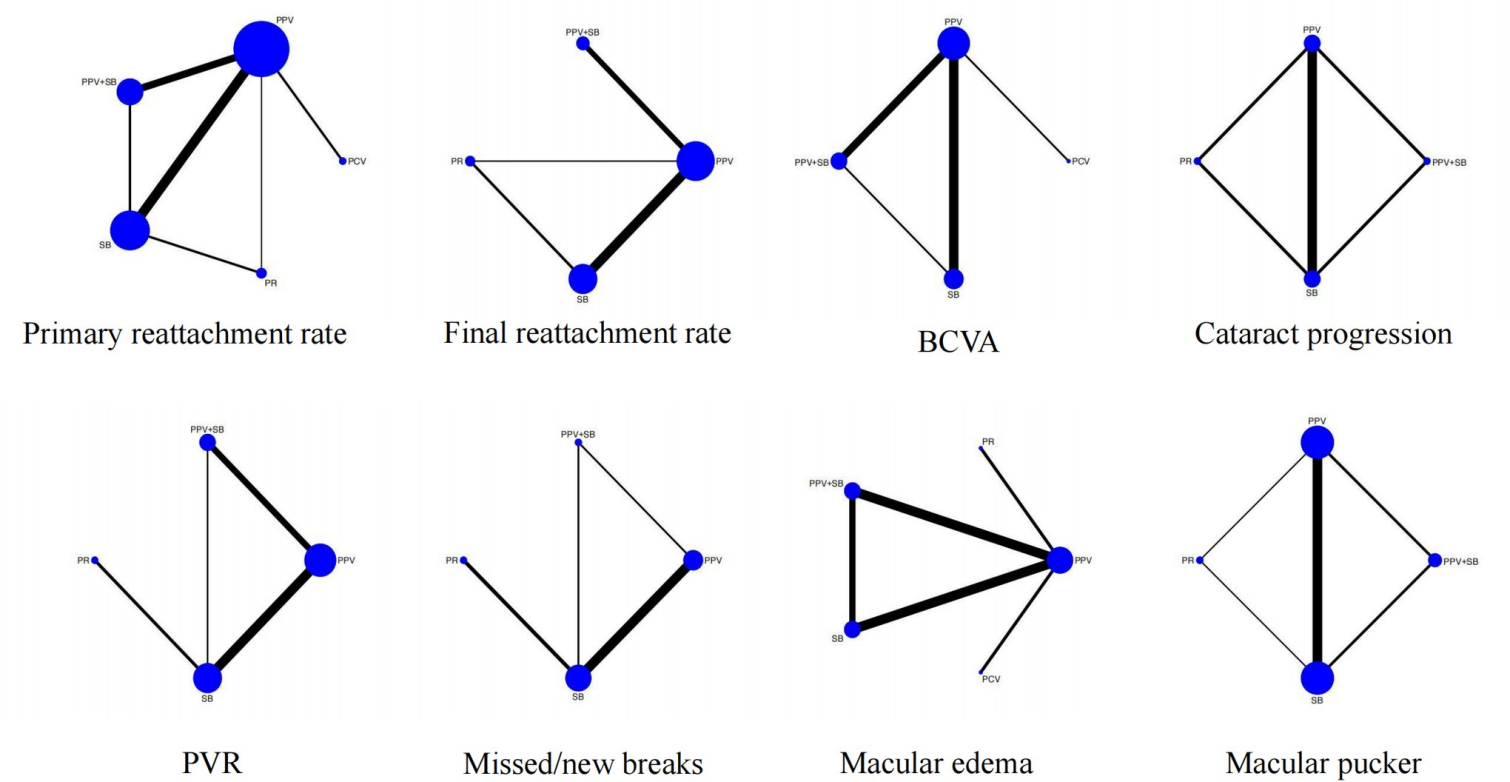
Network plots. The above figure shows the efficacy and safety of different interventions for the treatment of rhegmatogenous retinal detachment. Node size and line thickness were weighted based on the number of studies evaluating each treatment and direct comparisons, respectively.

The network plot shows that all included interventions were well-connected. In all outcomes, it shows that PPV and SB had the largest sample size (the biggest node), and most studies have compared PPV with SB (the width of line) (Fig 2).

### Network meta-analysis

#### Retinal reattachment rate

**Fig 3:**
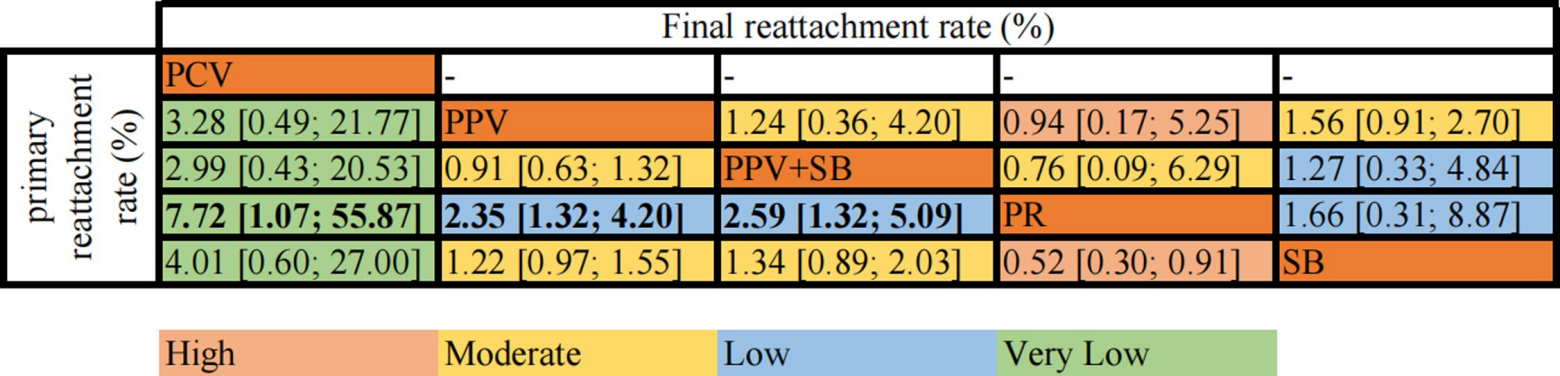
Net league table (primary reattachment rate and final reattachment rate). The above table shows the results of network meta-analysis of the anatomic success and the corresponding GRADE (Recommendation, Evaluation, Development, and Evaluation Grade) evidence certainty. The values in the table represent OR values with 95% confidence intervals, each of which compares the two interventions. Values in bold indicate a statistically significant treatment effect. Abbreviation: PPV, pars plana vitrectomy; SB, scleral buckling; PR, pneumatic retinopathy; PCV, pars plana vitrectomy combined with phacomulsification.

A total of 19 studies assessed Single-operation retinal reattachment rate (2512 eyes) (Fig 3). There were 18 two-arm studies and 1 three-arm study. Pooled network OR values indicate that compared with PR, PCV (OR = 7.72, 95% CI [1.07; 55.87], very low certainty), PPV+SB (OR = 2.59, 95% CI [1.32; 4.20], low certainty), PPV (OR = 2.35, 95% CI [1.32; 4.20], low certainty), SB (OR = 0.52, 95% [0.30; 0.91], high certainty) were associated with the higher primary retinal reattachment rate. Notably, we discover that the combined procedure did not show better results compared to SB (OR = 1.21, 95% CI [0.64; 2.28], moderate certainty), or PPV (OR = 0.91, 95% CI [0.63; 1.32], moderate certainty) alone. The SUCRA probability ranking showed that PCV (93%) had the greatest effect on single-operation retinal reattachment rate, followed by PPV+SB (68%), PPV (59%), SB (29%) and PR (1%).

Fourteen studies (2204 eyes) evaluated the final retinal attachment rate for PPV, SB, PPV+SB, and PR. (Fig 3) The results showed no significant differences between the four surgical modalities. According to SUCRA, PPV (71%) had the highest probability of effectiveness, while SB had the lowest (27%).

#### Best-corrected visual acuity at postoperative 6-month

**Fig 4:**
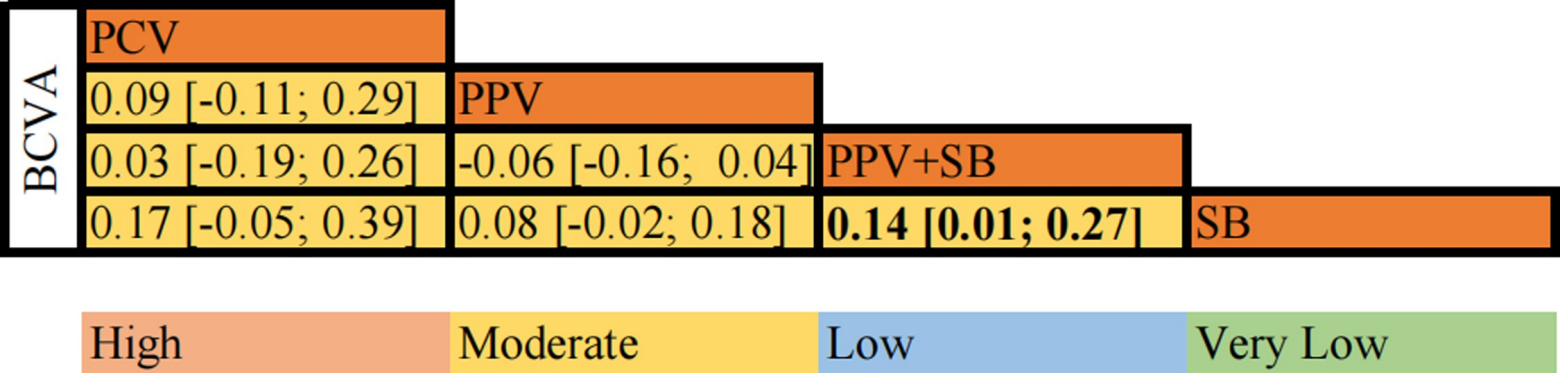
Net league table (best-corrected visual acuity at postoperative 6-month). Note: The above table shows the results of the network meta-analysis of BCVA at 6-month of postoperative and the corresponding GRADE (Recommendation, Assessment, Development, and Evaluation Grade) evidence certainty. The values in the table represent mean difference (MD) with 95% confidence intervals, each of which compares the two interventions. Values in bold indicate a statistically significant treatment effect. Abbreviation: PPV, pars plana vitrectomy; SB, scleral buckling; PCV, pars plana vitrectomy combined with phacomulsification.

A total of 9 RCTs with 904 eyes were included in our BCVA meta-analysis at postoperative 6 months. (Fig 4) We compared and ranked the effects of four surgical options other than PR. The findings showed that SB (MD = 0.14, 95% CI [0.01; 0.27], moderate certainty) had significantly better visual acuity outcomes than combined with PPV, while the other surgical options were statistically comparable. The SUCRA probability ranking showed that PCV (81%) were associated with highest effect on BCVA, followed by PPV+SB (77%), PPV (37%), SB (6%).

#### Postoperative cataract progression

**Fig 5:**
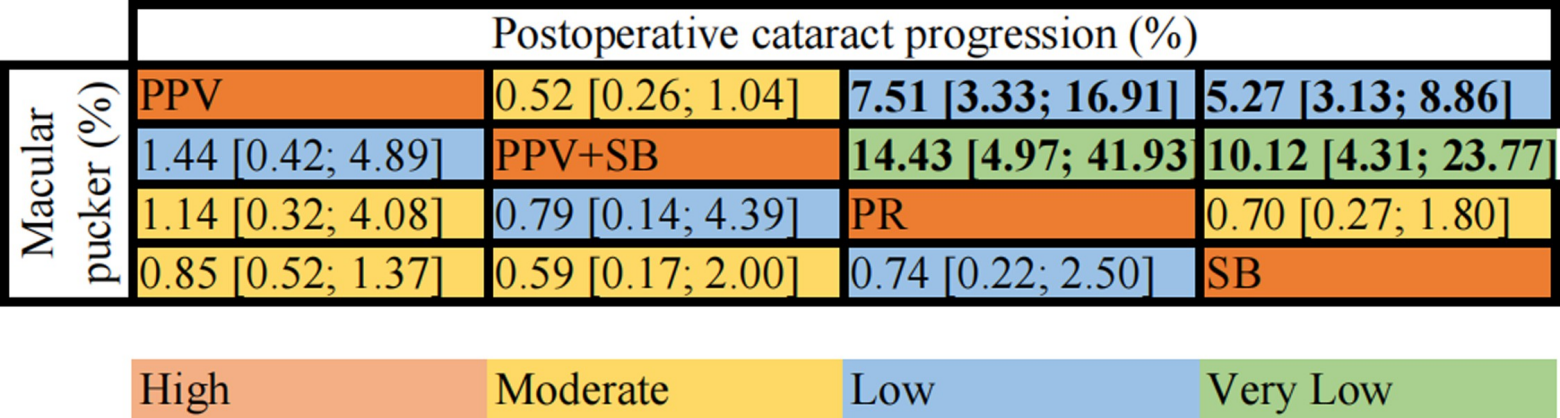
Net league table (postoperative cataract progression and macular pucker). Note: The above table shows the results of network meta-analysis of the Incidence of postoperative cataracts and macular pucker and the corresponding GRADE (Recommendation, Evaluation, Development, and Evaluation Grade) evidence certainty. The values in the table represent OR values with 95% confidence intervals, each of which compares the two interventions. Values in bold indicate a statistically significant treatment effect. Abbreviation: PPV, pars plana vitrectomy; SB, scleral buckling; PR, pneumatic retinopathy.

7 studies with 1028 eyes were analyzed for the postoperative cataract progression. (Fig 5) Compared with SB, PPV+SB (OR = 10.12, 95% CI [4.31; 23.77], very low certainty) and PPV (OR = 5.27, 95% CI [3.13; 8.86], moderate certainty) had higher incidence rate of postoperative cataract progression. And compared PR, PPV+SB (OR = 14.43, 95% CI [4.97; 41.93], very low certainty) and PPV (OR = 7.51, 95% CI [3.33; 16.91], low certainty) and also showed a higher rate of incidence of cataract progression. There was no statistically significant difference between PPV (OR = 0.52, 95% CI [0.26; 1.04], moderate certainty) and combined surgery in terms of postoperative cataract incidence. At the top of the SUCRA, probability ranking were PPV+SB (65%) and SB (61%).

#### Other results

S10 File shows the comparison of various surgical options for postoperative complications (the incidence of postoperative macular edema, macular pucker, missed/new breaks, and PVR) in patients with RRD, the findings indicate that all interventions yielded no statistically difference.

### Inconsistency analyses

The global and local inconsistency test was to determine the consistency level. All fitted models converged well, and there was no evidence to indicate statistical inconsistency in our NMA. (S8 File) Besides, the results of the network meta-regression on the seven covariates showed no subgroup effects in all analysis which further confirming the robustness of the current results. (S9 File) Given that there were fewer than 10 studies included in each pair of direct comparisons, we did not use funnel plots to assess publication bias.

## Discussion

Based on 19 RCTs with 2589 eyes randomly assigned to five types of intervention, this NMA summaries the efficacy and safety of PPV, SB, PR, PCV and PPV+SB in the treatment of patients with RRD. Our results showed that PR demonstrated worse initial successful rate and similar final successful rate. That’ s what we expect. The delayed reabsorption of subretinal fluid and poor compliance of some patients to keep in the proper position may be the major causes of operation failures [1]. Due to a lack of available data, the vision of patients with PR was unable to analyze. But an RCT of 176 patients reported that PR had better visual acuity and lower vertical distortion compared with PPV alone [28]. More high-quality RCTs are needed to validate this result. In terms of postoperative complications, we found a significant reduction in the incidence of postoperative cataracts with PR compared to PPV and PPV+SB, and there was no difference in others. This result is similar to that of previous studies. Hiller et al. reported a cataract incidence within 12 months of surgery in patients with PPV and PR, with PPV being 65% versus PR being only 16% [28].

In addition, the findings also suggested that combined surgery lacks advantage compared to primary SB and PPV. Combined surgery is currently used in patients at high risk of re-detachment, such as RRD with multiple inferior retinal breaks and inferior PVR [29]. Nevertheless, a meta-analysis by Jonathan et al. of patients with RRD combined with inferior breaks demonstrated the lack of benefit of adding SB to PPV compared to SB or PPV alone [30]. Although a previous study demonstrated that combined procedures showed a higher primary reattachment rate compared to PPV alone [31], but it is noteworthy that up to 84% of the included studies were observational which may cause some bias in the results. The results of meta-regression analysis suggested that differences in PVR grade at baseline did not influence our results. However, since patients with PVR grade C or higher were present in only two of the studies we included, not much is known about the use of combined surgery in this context [32,33]. A population of patients with RRD combined with PVR grade C or above may be needed in the future to study different treatment approaches. In terms of BCVA, we found that patients who underwent SB had significantly better visual acuity at six months than those who underwent combined surgery. There was none of previous literature supporting for this finding. However, the RCT by Moradian et al. [14] found that patients in the SB group improved their vision faster than the SB+PPV group at 12 months. A comparative intervention study also confirmed this result [34]. Furthermore, in terms of postoperative complications, we found no difference except for the incidence of cataracts. Available evidence suggests that PPV and combined surgery promotes cataract development in postoperative patients compared to PR and SB. This is consistent with previous findings and may be related to intraocular surgery and with endotamponade agents used after PPV [29,35].

### Compared with other studies

To our knowledge, this is the first network meta-analysis comparing five types of RRD repair techniques. Previous studies [36–41] have reported the effects of surgery in patients with RRD. However, their studies only made direct comparisons and lacked pooled analysis of indirect evidence. For example, Popovic et al. [40] studied the efficacy and safety of PR, PPV, SB and PPV combined SB in the treatment of RRD patients, but they were unable to analyze PR versus PPV and PPV+SB due to a lack of data during the analysis process, resulting in uneveness results. By integrating direct and indirect evidence, our analysis provides vitreoretinal surgeons with quantitative comparisons not found in prior reviews. Previous meta-analyses included more retrospective studies, which may be a greater risk of indication confusion and uncertainty quality of evidence. For example, Bonnar et al. [30] included fifteen studies comparing the efficacy of SB, PPV, and combination surgery in RRD patients with inferior breaks, nine of these studies were retrospective studies so that it may overstate or understate the level of evidence. Besides, we assessed the quality of the evidence and presented the results using coloured shading in the list to indicate the GRADE results, making the strength of the evidence more visual.

### Strengths and limitations

Compared with traditional meta-analysis, one substantial advantage of network meta-analysis is the ability to consider comparisons from all interventions in the study, including evidence from direct and indirect comparisons [42–44].

However, there are some limitations to our study. First, Differences between patients may affect the final results. Taking into account the possible heterogeneity between some of the comparisons, we performed regression analyses of covariates such as lens status, PVR grade, severity of RRD, age, year of publication, sample size and so on to explore whether the covariates influenced the final results. Nevertheless, other factors such as macular status, number and location of breaks, size of detachments, and vitreous hemorrhage, could not be analyzed due to incomplete reporting of the original studies, which may have led to significant confounding bias. Second, surgeon proficiency, intraoperative fillers (e.g., air, silicone oil, etc.) and device selection (e.g., different gauge valved trocars and types of silicone elements) may also contribute to some degree of bias. Third, we included fewer patients with PR because of lacking original studies, perhaps resulting in unstable results. Besides, due to the small number of eligible outcomes and time points, there is a lack of available data for several outcomes, resulting in some results that could not be analyzed. Finally, we found some primary studies to be at high risk of bias, mainly due to uncertainty about the randomization process, which affected the overall level of evidence.

### Future research

Most current studies on RRD surgery are observational, and high-quality RCTs are still urgently needed. Second, data on baseline and outcomes in the RCTs remain incomplete, such as surgeon’s proficiency, number and location of lacunae, and choice of filler, more comprehensive and accurate data reporting is needed for future studies. In a word, researchers should strictly adhere to Consolidated Standards of Reporting Trials to perform high-quality RCTs and further explore the efficacy and safety of different surgical options for the treatment of RRD.

## Conclusion

In summary, this network meta-analysis found PR appear to associate with a lower rate of primary reattachment rate and postoperative cataract progression. Besides, there are no anatomical, functional, or postoperative complication advantages of the combined procedure over PPV or SB alone. It was noting that significant evidence limitations remain, necessitating improved comparative effectiveness trials to further optimize RRD surgery guidelines.

## Supporting information

S1 File Search strategy. (DOCX)

S2 File Details on the measurement indicators. (DOCX)

S3 File Data management and details on the missing mean and standard deviations. (DOCX)

S4 File The list of eligible studies. (DOCX)

S5 File Basic characteristics of the included studies. (DOCX)

S6 File The evidence findings for all comparisons. (DOCX)

S7 File SUCRA values (%) for all outcomes. (DOCX)

S8 File Node-splitting test for inconsistency. (DOCX)

S9 File Network meta-regression results for abstinence rate. (DOCX)

S10 File NMA results with corresponding certainty of evidence. (DOCX)

S11 File Network plots. (DOCX)

S12 File Traffic light of risk of bias of included studies. (DOCX)

S13 File Summary graph of risk of bias of included studies. (DOCX)

S14 File PRISMA NMA checklist. (DOCX)

S15 File List of excluded studies during full-text screening and reasons for their exclusions. (DOCX)

S1 Table The outcome data extracted from the primary research. (XLSX)

S2 Table Records excluded on the basis of title or abstract and the reasons. (XLSX)
