# Supplementary material for: Surgical managements for rhegmatogenous retinal detachment: A network meta-analysis of randomized controlled trial
Source: PLoS One. 2024 Nov 14;19(11):e0310859. doi: 10.1371/journal.pone.0310859 (PMC11563380; doi:10.1371/journal.pone.0310859)
Supplement: S1 File — (DOCX) [file pone.0310859.s001.docx]

**S1 File: Search Strategies**

| # | **PubMed** |
| --- | --- |
| 1 | "Retinal Detachment/diagnosis"[Mesh] OR "Retinal Detachment/etiology"[Mesh] OR "Retinal Detachment/surgery"[Mesh] OR Detachment, Retinal[Title/Abstract] OR Detachment, Retinal[Title/Abstract] OR Detachments, Retinal[Title/Abstract] OR Retinal Detachments[Title/Abstract] OR Retinal Pigment Epithelial Detachment[Title/Abstract] OR rhegmatogenous retinal detachment[Title/Abstract] |
| 2 | “Scleral Buckling/adverse effects"[Mesh] OR "Scleral Buckling/methods"[Mesh] OR Buckling, Scleral[Title/Abstract] OR Bucklings, Scleral[Title/Abstract] OR Scleral Bucklings [Title/Abstract] OR Scleral buckle[Title/Abstract] OR Buckle, Scleral[Title/Abstract] OR "Vitrectomy"[Mesh] OR Vitrectomies[Title/Abstract] OR pars plana vitrectomy[Title/Abstract] OR pneumatic retinopexy[Title/Abstract] OR pneumoretinopexy[Title/Abstract] OR “Phacoemulsification”[Mesh] OR Phacoemulsification[Title/Abstract] OR Phacomulsifications[Title/Abstract] |
| 3 | "Clinical Trials as Topic"[Mesh] OR "Controlled Clinical Trial" [Publication Type] OR "Clinical Trial, Phase IV" [Publication Type] OR "Clinical Trial, Phase III" [Publication Type] OR "Clinical Trial, Phase II" [Publication Type] OR "Clinical Trial, Phase I" [Publication Type] OR "Adaptive Clinical Trial" [Publication Type] OR "Randomized Controlled Trial" [Publication Type] OR "Clinical Trial" [Publication Type] OR Intervention Study[Title/Abstract] OR Clinical Trial as Topic[Title/Abstract] OR Clinical Trial, Phase 4[Title/Abstract] OR Clinical Trial, Phase 3[Title/Abstract] OR Clinical Trial, Phase 2[Title/Abstract] OR Clinical Trial, Phase 1[Title/Abstract] OR Single-Blind Method[Mesh] OR Double-Blind Method[Mesh] OR random*[Title/Abstract] OR blind*[Title/Abstract] OR singleblind*[Title/Abstract] OR doubleblind*[Title/Abstract] OR tripleblind*[Title/Abstract] |
| 4 | #1 AND #2 AND #3 |

| # | **Web of Science** |
| --- | --- |
| 1 | TS=（Retinal Detachment OR Retinal Detachments OR Retinal Pigment Epithelial Detachment OR rhegmatogenous retinal detachment） |
| 2 | TS=（Scleral Buckling OR Scleral Bucklings OR Vitrectomy OR Vitrectomies OR pars plana vitrectomy OR pneumatic retinopexy OR pneumoretinopexy OR phacoemulsification OR phacoemulsifications） |
| 3 | TS= (randomized controlled trial OR Clinical trial OR Controlled Clinical trial OR random* OR blind* OR singleblind* OR doubleblind* OR tripleblind* OR trebleblind*) |
| 4 | #1 AND #2 AND #3 |

| # | **Cochrane Library** |
| --- | --- |
| 1 | MeSH descriptor: [Retinal Detachment] explode all trees |
| 2 | (Detachment, Retinal OR Detachment, Retinal OR Detachments, Retinal OR Retinal Detachments OR Retinal Pigment Epithelial Detachment OR rhegmatogenous retinal detachment):ti,ab,kw (Word variations have been searched) |
| 3 | MeSH descriptor: [Scleral Buckling] explode all trees |
| 4 | MeSH descriptor: [Vitrectomy] explode all trees |
| 5 | MeSH descriptor: [phacoemulsification] explode all trees |
| 6 | (Buckling, Scleral OR Bucklings, Scleral OR Scleral Bucklings OR Vitrectomy OR Vitrectomies OR pars plana vitrectomy OR pneumatic retinopexy OR pneumoretinopexy OR phacoemulsification OR phacoemulsifications):ti,ab,kw (Word variations have been searched) |
| 7 | #1 OR #2 |
| 8 | #3 OR #4 OR #5 OR #6 |
| 9 | #7 AND #8 |

| # | **Embase** |
| --- | --- |
| 1 | 'retinal detachment'/exp OR 'retinal detachment' OR 'retinal detachments' OR 'retinal pigment epithelial detachment'/exp OR 'retinal pigment epithelial detachment' OR 'rhegmatogenous retinal detachment'/exp OR 'rhegmatogenous retinal detachment' |
| 2 | 'scleral buckling' OR 'scleral bucklings' OR 'vitrectomy' OR 'vitrectomies' OR 'pars plana vitrectomy' OR 'pneumatic retinopexy' OR ' pneumoretinopexy ' OR ' phacoemulsification ' |
| 3 | 'multicenter study (topic)'/exp OR 'phase 2 clinical trial (topic)'/exp OR 'phase 3 clinical trial (topic)'/exp OR 'phase 4 clinical trial (topic)'/exp OR 'controlled clinical trial (topic)'/exp OR 'randomized controlled trial (topic)'/exp OR 'single blind procedure'/exp OR 'double blind procedure'/exp OR random*:ab,ti OR blind*:ab,ti OR singleblind*:ab,ti OR doubleblind*:ab,ti OR trebleblind*:ab,ti OR tripleblind*:ab,ti |
| 4 | #1 AND #2 AND #3 |
