# Supplementary material for: Surgical managements for rhegmatogenous retinal detachment: A network meta-analysis of randomized controlled trial
Source: PLoS One. 2024 Nov 14;19(11):e0310859. doi: 10.1371/journal.pone.0310859 (PMC11563380; doi:10.1371/journal.pone.0310859)
Supplement: S2 File — (DOCX) [file pone.0310859.s002.docx]

**S2 File: Details on the measurement indicators**

Network plot: The plot of a network of interventions is a visual presentation of the evidence base and provides a concise description of its characteristics. The amount of available information can be presented by ‘weighting’ the nodes and lines: different node sizes representing the number of studies of available interventions and line thicknesses indicating the frequency of available direct comparisons between pairs of interventions.^1^

Surface under the cumulative ranking (SUCRA) probability ranking：SUCRA allows ranking the interventions on a continuous 0-100% scale, with the higher value being more effective. For each treatment *j* out of the *a* competing interventions, we calculate the *a* vector of cumulative probabilities cum *_j, b_* to be among the *b* best treatments, *b* = 1…*a*. The surface below the cumulative step function for treatment *j* is: ^2^

SUCRA_j_ =
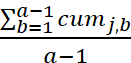


League table: Net league table used to show a concise comparison of all possible pairings between interventions.^3^ The values in each table represent SMD with 95% confidence interval, and each SMD value is the result of comparing two interventions. For a series of SMDs, each SMD is the result of a straight-line comparison between the interventions of the high and low steps.

Reference

1. Chaimani A, Higgins JP, Mavridis D, Spyridonos P, Salanti G. Graphical tools for network meta-analysis in STATA. *PLoS One*. 2013;8(10): e76654. Published 2013 Oct 3. doi:10.1371/journal.pone.0076654
2. Salanti G, Ades AE, Ioannidis JP. Graphical methods and numerical summaries for presenting results from multiple-treatment meta-analysis: an overview and tutorial. *J Clin Epidemiol*. 2011;64(2):163-171. doi:10.1016/j.jclinepi.2010.03.016
3. Hutton B, Salanti G, Caldwell DM, et al. The PRISMA extension statement for reporting of systematic reviews incorporating network meta-analyses of health care interventions: checklist and explanations. *Ann Intern Med*. 2015;162(11):777-784. doi:10.7326/M14-2385
