# Supplementary material for: Surgical managements for rhegmatogenous retinal detachment: A network meta-analysis of randomized controlled trial
Source: PLoS One. 2024 Nov 14;19(11):e0310859. doi: 10.1371/journal.pone.0310859 (PMC11563380; doi:10.1371/journal.pone.0310859)
Supplement: S3 File — (DOCX) [file pone.0310859.s003.docx]

**S3 File. Data management and details on the missing mean and standard deviations**

**Methods for imputation of missing mean**

We used the method proposed by Wan and colleagues^1^ to impute a missing mean value for studies reported the median, lower quartile, and upper quartile summary statistics.

1. Wan X, Wang W, Liu J, et al. Estimating the sample mean and standard deviation from the sample size, median, range and/or interquartile range. BMC Med Res Methodol, 2014, 14: 135.

**Methods for imputation of missing** **standard deviations**

We used published standard deviations (SDs), where available. When SDs were not presented, we estimated SDs from standard errors, P values or confidence interval (CIs) according to the recommendations of the Cochrane Handbook for Systematic Reviews^1^.


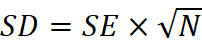


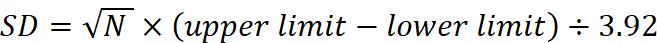


If studies reported medians and interquartile ranges (IQRs), we used median to impute the missing mean and calculated SDs according to the method proposed by Wan and colleagues^2^. We also estimated SDs from graphs when they were missing in tables or in text. If none of these options are viable, we imputed the missing SDs using pooled SDs from other studies included in our meta-analysis following the formula below:^3^


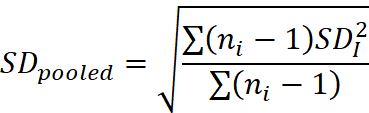


1. Higgins JPT, Deeks JJ (editors). Chapter 7: Selecting studies and collecting data. In: Higgins JPT, Green S (editors), Cochrane Handbook for Systematic Reviews of Interventions. Version 5.1.0 [updated March 2011]. The Cochrane Collaboration, 2011. Available from www.cochrane-handbook.org.
2. Wan X, Wang W, Liu J, et al. Estimating the sample mean and standard deviation from the sample size, median, range and/or interquartile range. BMC Med Res Methodol, 2014, 14: 135.
3. Furukawa T A, Barbui C, Cipriani A, et al. Imputing missing standard deviations in meta-analyses can provide accurate results. Journal of Clinical Epidemiology, 2006, 59(1): 7-10.
