# Supplementary material for: Surgical managements for rhegmatogenous retinal detachment: A network meta-analysis of randomized controlled trial
Source: PLoS One. 2024 Nov 14;19(11):e0310859. doi: 10.1371/journal.pone.0310859 (PMC11563380; doi:10.1371/journal.pone.0310859)
Supplement: S4 File — (DOCX) [file pone.0310859.s004.docx]

**S4 File: The list of eligible studies.**

**1.** Tornambe, P. E., Hilton, G. F., Poliner, L. S., Brinton, D. A., Flood, T. P., Orth, D. H., ... & Tiedeman, J. S. (1989). Pneumatic retinopexy: a multicenter randomized controlled clinical trial comparing pneumatic retinopexy with scleral buckling. *Ophthalmology*, *96*(6), 772-784.

**2.** Mulvihill, A., Fulcher, T., Datta, V., & Acheson, R. (1996). Pneumatic retinopexy versus scleral buckling: a randomised controlled trial. *Irish Journal of medical science*, *165*, 274-277.

3. Heimann, H., Bartz-Schmidt, K. U., Bornfeld, N., Weiss, C., Hilgers, R. D., Foerster, M. H., & Scleral Buckling versus Primary Vitrectomy in Rhegmatogenous Retinal Detachment Study Group. (2007). Scleral buckling versus primary vitrectomy in rhegmatogenous retinal detachment: a prospective randomized multicenter clinical study. *Ophthalmology*, *114*(12), 2142-2154.

4. Brazitikos, P. D., Androudi, S., Christen, W. G., & Stangos, N. T. (2005). Primary pars plana vitrectomy versus scleral buckle surgery for the treatment of pseudophakic retinal detachment: a randomized clinical trial. *Retina*, *25*(8), 957-964.

5. Sharma, Y. R., Karunanithi, S., Azad, R. V., Vohra, R., Pal, N., Singh, D. V., & Chandra, P. (2005). Functional and anatomic outcome of scleral buckling versus primary vitrectomy in pseudophakic retinal detachment. *Acta Ophthalmologica Scandinavica*, *83*(3), 293-297.

6. KHAN, R., KHAN, A. A., & CH, N. A. (2019). Scleral buckling and pars plana vitrectomy versus pars plana vitrectomy alone in the treatment of rhegmatogenous retinal detachment with inferior break. *Age (years)*, *42*, 12-5.

7. Mehboob, M. A. R. I. A., Ghani, M. U., Khan, A. S. M. A., & Imran, M. (2018). Scleral buckling and pars plana vitrectomy versus vitrectomy alone for primary repair of rhegmatogenous retinal detachment. *Pakistan J Med Health Sci*, *12*, 716-720.

8. Mora, P., Favilla, S., Calzetti, G., Berselli, G., Benatti, L., Carta, A., ... & Tedesco, S. A. (2021). Parsplana vitrectomy alone versus parsplana vitrectomy combined with phacoemulsification for the treatment of rhegmatogenous retinal detachment: a randomized study. *BMC ophthalmology*, *21*, 1-7.

9. Zhao, X., Huang, L., Lyu, C., Liu, B., Ma, W., Deng, X., ... & Lu, L. (2020). Comparison between releasable scleral buckling and vitrectomy in patients with phakic primary rhegmatogenous retinal detachment. *Retina (Philadelphia, Pa.)*, *40*(1), 33.

10. Hillier, R. J., Felfeli, T., Berger, A. R., Wong, D. T., Altomare, F., Dai, D., ... & Muni, R. H. (2019). The pneumatic retinopexy versus vitrectomy for the management of primary rhegmatogenous retinal detachment outcomes randomized trial (PIVOT). *Ophthalmology*, *126*(4), 531-539.

11. Walter, P., Hellmich, M., Baumgarten, S., Schiller, P., Limburg, E., Agostini, H., ... & Mazinani, B. (2017). Vitrectomy with and without encircling band for pseudophakic retinal detachment: VIPER Study Report No 2—main results. *British Journal of Ophthalmology*, *101*(6), 712-718.

12. Moradian, S., Ahmadieh, H., Faghihi, H., Ramezani, A., Entezari, M., Banaee, T., ... & Yasseri, M. (2016). Comparison of four surgical techniques for management of pseudophakic and aphakic retinal detachment: a multicenter clinical trial. *Graefe's Archive for Clinical and Experimental Ophthalmology*, *254*, 1743-1751.

13. Falkner‐Radler, C. I., Graf, A., & Binder, S. (2015). Vitrectomy combined with endolaser or an encircling scleral buckle in primary retinal detachment surgery: a pilot study. *Acta ophthalmologica*, *93*(5), 464-469.

14. Romano, M. R., Angi, M., Valldeperas, X., Costagliola, C., & Vinciguerra, P. (2011). Twenty-three–gauge pars plana vitrectomy, densiron-68, and 360 endolaser versus combined 20-gauge pars plana vitrectomy, scleral buckle, and SF6 for pseudophakic retinal detachment with inferior retinal breaks. *Retina*, *31*(4), 686-691.

15. Koriyama, M., Nishimura, T., Matsubara, T., Taomoto, M., Takahashi, K., & Matsumura, M. (2007). Prospective study comparing the effectiveness of scleral buckling to vitreous surgery for rhegmatogenous retinal detachment. *Japanese journal of ophthalmology*, *51*, 360-367.

16. Ahmadieh H, Moradian S, Faghihi H, et al. Anatomic and visual outcomes of scleral buckling versus primary vitrectomy in pseudophakic and aphakic retinal detachment: six-month follow-up results of a single operation--report no. 1. *Ophthalmology*. 2005;112(8):1421-1429. doi:10.1016/j.ophtha.2005.02.018

17. Tewari, H. K., Kedar, S., Kumar, A., Garg, S. P., & Verma, L. K. (2003). Comparison of scleral buckling with combined scleral buckling and pars plana vitrectomy in the management of rhegmatogenous retinal detachment with unseen retinal breaks: Clinical Research. *Clinical & experimental ophthalmology*, *31*(5), 403-407.

18. Dahab, A. A., Helmy, Y. A., Khattab, A. M., Abdelhakim, M. A., & Hamza, H. S. (2020). Vitrectomy and silicone oil tamponade with and without phacoemulsification in the management of rhegmatogenous retinal detachment: A comparative study. *African Vision and Eye Health*, *79*(1), 1-8.

19. Azad, R. V., Chanana, B., Sharma, Y. R., & Vohra, R. (2007). Primary vitrectomy versus conventional retinal detachment surgery in phakic rhegmatogenous retinal detachment. *Acta Ophthalmologica Scandinavica*, *85*(5), 540-545.
