# Supplementary material for: Surgical managements for rhegmatogenous retinal detachment: A network meta-analysis of randomized controlled trial
Source: PLoS One. 2024 Nov 14;19(11):e0310859. doi: 10.1371/journal.pone.0310859 (PMC11563380; doi:10.1371/journal.pone.0310859)
Supplement: S5 File — (DOCX) [file pone.0310859.s005.docx]

**S5 File. Basic characteristics of the included studies**

| Study | Surgery | Location | Eyes, n | Age, Years (μ ± σ) | Sex, n (%) males | Study eye, n (%) OD | Phakic, n (%) | Aphakic, n (%) | Pseudophakic, n (%) | BCVA, Log Mar (μ ± σ) | IOP, mmHg (μ ± σ) | Macular off, n (%) | Inferior breaks, n (%) | Extension of RD (quadrant), (μ ± σ) | Lattice degeneration, n (~~%)~~ | VH, n (%) | PVR Grade C or Worse, n(%) | Target F/U, months |
| --- | --- | --- | --- | --- | --- | --- | --- | --- | --- | --- | --- | --- | --- | --- | --- | --- | --- | --- |
| Tornambe,1989 | SB | American | 95 | -- | 65(68) | -- | 51(54) | 10(10) | 34(36) | -- | -- | -- | -- | -- | -- | 5(5) | 0 | 6 |
|  | PR |  | 103 | -- | 64(62) | -- | 57(55) | 10(10) | 36(35) | -- | -- | -- | -- | -- | -- | 9(9) | 0 | 6 |
| Mulvihill A,1996 | PR | Iran | 10 | -- | -- | -- | -- | -- | -- | -- | -- | -- | 0 | -- | -- | -- | 0 | 16 |
|  | SB |  | 10 | -- | -- | -- | -- | -- | -- | -- | -- | -- | 0 | -- | -- | -- | 0 | 16 |
| Heimann,2007 | SB | Germany | 342 | 63.3 ± 11.1 | 214(62.6) | -- | 209(61.1) | -- | 133(38.9) | -- | 14.5 ± 3.9 | 221(64.6) | 0 | -- | -- | -- | 0 | 12 |
|  | PPV |  | 339 | 61.6 ± 10.8 | 226(66.7) | -- | 207(61.1) | -- | 132(38.9) | -- | 14.5 ± 3.2 | 212(62.5) | 0 | -- | -- | -- | 0 | 12 |
| Brazitikos,2005 | SB | Greece | 75 | 71.01 ± 8.13 | 42(56.0) | -- | 0 | 0 | 75(100) | 1.09 ± 0.46 | -- | 60(80) | -- | -- | 0 | 0 | 0 | 12 |
|  | PPV |  | 75 | 73.01 ± 8.57 | 45(60) | -- | 0 | 0 | 75(100) | 0.98 ± 0.52 | -- | 53(71) | -- | -- | 0 | 0 | 0 | 12 |
| Sharma,2005 | SB | India | 25 | 56.8 ± 12 | 20(80) | -- | 0 | 0 | 25(100) | 0.04 ± 0.05 | 12.72 ± 4.81 | -- | 9(38) | -- | -- | -- | 0 | 6 |
|  | PPV |  | 25 | 58.28 ± 9.14 | 20(80) | -- | 0 | 0 | 25(100) | 0.06 ± 0.10 | 11.52 ± 2.27 | -- | 7(28) | -- | -- | -- | 0 | 6 |
| Razaullah,2018 | PPV+SB | Pakistan | 15 | 42.5±12.6 | 18(60) | 17(56.7) | -- | -- | -- | 1.19±0.09 | -- | -- | 15(100) | -- | -- | -- | 0 | 6 |
|  | PPV |  | 15 | 42.6±12.7 | 20(66.7) | 16(53.3) | -- | -- | -- | 1.18±0.11 | -- | -- | 15(100) | -- | -- | -- | 0 | 6 |
| Mehboob,2018 | PPV | Pakistan | 100 | 54.8 ± 5.64 | 82(82) | -- | 100(100) | 0 | 0 | -- | -- | 52(52) | 14(14) | 4.81 ± 2.26(clock hours) | 6(6) | -- | 0 | 6 |
|  | PPV+SB |  | 100 | 55.6 ± 4.51 | 63(63) | -- | 100(100) | 0 | 0 | -- | -- | 73(73) | 57(57) | 6.48 ± 2.09 | 19(19) | -- | 0 | 6 |
| Mora,2021 | PPV | Italy | 29 | 54 ± 5 | 19(66) | -- | 29(100) | 0 | 0 | -- | 12.9 ± 3.5 | 17(58.62) | 0 | -- | 0 | -- | 0 | 6 |
|  | PCV |  | 30 | 56 ± 7 | 17(57) | -- | 30(100) | 0 | 0 | -- | 14.1 ± 2.8 | 20(66.67) | 0 | -- | 0 | -- | 0 | 6 |
| Zhao,2020 | PPV | China | 55 | 48.54 ± 11.77 | 25(45.45) | -- | 55(100) | -- | -- | 1.41 ± 0.96 | 12.54 ± 2.52 | 37(67.27) | 16(29.09) | 2.88 ± 0.77 | -- | -- | 0 | 12 |
|  | SB |  | 55 | 42.18 ± 15.50 | 32(58.18) | -- | 55(100) | -- | -- | 1.26 ± 0.78 | 11.26 ± 2.75 | 33(60.00) | 18(32.73) | 2.76 ± 0.72 | -- | -- | 0 | 12 |
| Hillier,2019 | PR | Canada | 88 | 60.7 ± 10.1 | 61(69) | 54(61) | 57(65) | -- | 31(35) | -- | -- | 44(50) | 0 | 1.81 ± 0.79 | -- | 0 | 0 | 12 |
|  | PPV |  | 88 | 60.3 ± 7.8 | 55(62) | 39(44) | 62(70) | -- | 26(30) | -- | -- | 44(50) | 0 | 1.65 ± 0.73 | -- | 0 | 0 | 12 |
| Walter,2016 | PPV+SB | Germany | 100 | 65 ± 10 | 70(70) | 51(51) | 0 | 0 | 100(100) | 1.1 ± 0.23 | 14 ± 0.67 | -- | -- | -- | -- | 0 | 0 | 6 |
|  | PPV |  | 98 | 64 ± 10 | 71(72.4) | 55(56.1) | 0 | 0 | 98(100) | 0.8 ± 0.23 | 15 ± 0.83 | -- | -- | -- | -- | 1(1) | 0 | 6 |
| Moradian,2016 | SB | Iran | 50 | -- | -- | -- | 0 | -- | -- | 1.91 ± 0.75 | -- | 43(85.4) | 17(33.3) | -- | 0 | 0 | 0 | 12 |
|  | PPV |  | 51 | -- | -- | -- | 0 | -- | -- | 2 ± 0.75 | -- | 45(88.4) | 19(36.9) | -- | 0 | 0 | 0 | 12 |
|  | PPV+SB |  | 58 | -- | -- | -- | 0 | -- | -- | 1.8 ± 0.73 | -- | 51(87.7) | 22(38.4) | -- | 0 | 0 | 0 | 12 |
| Falkner,2015 | PPV | Austria | 30 | 63.77 ± 11.12 | 22(73.33) | 15(50) | 17(56.67) | 0 | 10(33.33) | 0.72 ± 0.48 | -- | 17(56.67) | 3(10.00) | 2.00 ± 0.59 | -- | -- | 0 | 6 |
|  | PPV+SB |  | 30 | 66.27 ± 9.20 | 19(63.33) | 13(43.33) | 13(33.33) | 0 | 20(66.67) | 0.88 ± 0.41 | -- | 22(73.33) | 10(34.48) | 0.40 ± 0.72 | -- | -- | 0 | 6 |
| Ramano,2011 | PPV | Italy | 44 | 57 ± 13 | 28(63.6) | -- | 0 | 0 | 44(100) | 0.90 ± 0.22 | 14.1 ± 2.4 | 32(72.7) | 44(100) | 2.1 ± 0.2 | -- | -- | 12(27.3) | 9 |
|  | PPV+SB |  | 38 | 61 ± 11 | 26(68.4) | -- | 0 | 0 | 38(100) | 0.78 ± 0.30 | 16.2 ± 1.8 | 24(63.2) | 38(100) | 1.8 ± 0.4 | -- | -- | 1(2.6) | 9 |
| Koriyama,2007 | SB | Japan | 23 | 59.4 ± 6.6 | 12(52.2) | -- | -- | -- | -- | 1.2 ± 0.89 | -- | -- | 9(39.1) | -- | -- | 0 | 0 | 36 |
|  | PPV |  | 23 | 61.1 ± 7.2 | 13(56.5) | -- | -- | -- | -- | 1.3 ± 0.98 | -- | -- | 8(34.8) | -- | -- | 0 | 0 | 36 |
| Ahmadieh,2005 | SB | Iran | 126 | 64.23 ± 11.34 | 76(60.3) | -- | 0 | 47(37.3) | 79(62.7) | 2.21 ± 0.67 | 9.72 ± 4.0 | 123（97.6） | -- | 2.95 ± 0.92 | -- | 9(7.2) | 0 | 6 |
|  | PPV |  | 99 | 60.63 ± 13.65 | 66(66.7) | -- | 0 | 34(34.4) | 65(65.7) | 2.37 ± 0.46 | 10.58 ± 3.73 | 97（98.0) | -- | 3.27 ± 3.02 | -- | 9(9.1) | 0 | 6 |
| Tewari,2003 | PPV+SB | India | 22 | 47.9 ± 18.13 | 13(59.1） | 9（40.9） | 4(18.2) | 9(40.9) | 7(31.8) | -- | -- | -- | -- | -- | -- | -- | 2(9.1) | 3 |
|  | SB |  | 22 | 48.95 ± 14.86 | 11(50） | 12（54.5） | 5(22.7) | 10(45.5) | 5(22.7) | -- | -- | -- | -- | -- | -- | -- | 1(4.5) | 3 |
| Youssef,2020 | PPV | Egypt | 20 | 57.6 ± 6.25 | 12(60) | -- | 20(100) | 0 | 0 | 2.3 ± 0.5 | 11.1 ± 3.00 | 9(45) | -- | -- | -- | -- | 0 | 3 |
|  | PCV |  | 20 | 56.8 ± 7.50 | 14(70) | -- | 20(100) | 0 | 0 | 2.4 ± 0.68 | 13.0 ± 1.50 | 12(60) | -- | -- | -- | -- | 0 | 3 |
| Azad,2007 | PPV | India | 30 | 41 ± 15 | 17(57) | 15(50) | 30(100) | 0 | 0 | 1.73 ± 0.91 | 11.7 ± 4.0 | 27(87) | -- | -- | -- | 0 | 0 | 6 |
|  | SB |  | 31 | 36 ± 16 | 23(74) | 18(58) | 31(100) | 0 | 0 | 1.43 ± 0.92 | 11.7 ± 2.5 | 25(83) | -- | -- | -- | 0 | 0 | 6 |

μ, mean; σ, standard deviation; --, data not available; BCVA, corrected distance visual acuity; F/U, follow-up; LogMAR, logarithm of the minimal angle of resolution; n, number; OD, right eye; PPV, pars plana vitrectomy; PVR, proliferative vitreoretinopathy; SB, scleral buckling; SB + PPV, scleral buckling in combination with pars plana vitrectomy; VH, vitreous hemorrhage.
