# Supplementary material for: Surgical managements for rhegmatogenous retinal detachment: A network meta-analysis of randomized controlled trial
Source: PLoS One. 2024 Nov 14;19(11):e0310859. doi: 10.1371/journal.pone.0310859 (PMC11563380; doi:10.1371/journal.pone.0310859)
Supplement: S6 File — (DOCX) [file pone.0310859.s006.docx]

**S6 File. The evidence findings for all comparisons**

**The evidence findings for all comparisons (Single-operation retinal reattachment rate)**

| **Certainty assessment** | | | | | | | **№ of Eyes** | | **Effect** | **Certainty** |
| --- | --- | --- | --- | --- | --- | --- | --- | --- | --- | --- |
| **№ of studies** | **Study design** | **Risk of bias** | **Inconsistency** | **Indirectness** | **Imprecision** | **Publication bias** | **Intervention-1** | **Intervention-2** | **OR with 95% CI** |  |
| **PPV vs SB** [3, 4, 5, 9, 12, 15, 16, 19] | | | | | | | | | | |
| **8** | RCT | Serious | Not serious | Not serious | Not serious | Undetected | 670 | 706 | 1.22 [0.97;1.55] | Moderate |
| **PPV vs PPV+SB** [6, 7, 11, 12, 13, 14] | | | | | | | | | | |
| **6** | RCT | Serious | Not serious | Not serious | Not serious | Undetected | 298 | 341 | 0.91 [0.63; 1.32] | Moderate |
| **PPV vs PCV** [8, 18] | | | | | | | | | | |
| **2** | RCT | Very serious | Not serious | Not serious | Very  serious | Undetected | 49 | 50 | 3.28 [0.49; 21.77] | Very low |
| **PPV+SB vs SB** [12, 17] | | | | | | | | | | |
| **2** | RCT | Serious | Not Serious | Not serious | Not serious | Undetected | 78 | 63 | 1.34 [0.89; 2.03] | Moderate |
| **PR vs SB** [1, 2] | | | | | | | | | | |
| **2** | RCT | Not serious | Not serious | Not serious | Not serious | Undetected | 113 | 105 | 0.52 [0.30; 0.91] | High |
| **PPV vs PR** [10] | | | | | | | | | | |
| **1** | RCT | Serious | Not serious | Not serious | Serious | Undetected | 73 | 78 | 2.35 [1.32; 4.20] | Low |
| **PPV+SB vs PCV** | | | | | | | | | | |
|  | RCT | Very  Serious | Not serious | Not serious | Very serious | Undetected |  |  | 2.99 [0.43; 20.53] | Very Low |
| **PR vs PCV** | | | | | | | | | | |
|  | RCT | Very serious | Not serious | Not  Serious | Serious | Undetected |  |  | 7.72 [1.07; 55.87] | Very  Low |
| **SB vs PCV** | | | | | | | | | | |
|  | RCT | Very serious | Not serious | Serious | Very  Serious | Undetected |  |  | 4.01 [0.60; 27.00] | Very Low |
| **PPV+SB vs PR** | | | | | | | | | | |
|  | RCT | Serious | Not Serious | Not serious | Serious | Undetected |  |  | 2.59 [1.32; 5.09] | Low |

**The evidence findings for all comparisons (Final retinal reattachment rate)**

| **Certainty assessment** | | | | | | | **№ of patients** | | **Effect** | **Certainty** |
| --- | --- | --- | --- | --- | --- | --- | --- | --- | --- | --- |
| **№ of studies** | **Study design** | **Risk of bias** | **Inconsistency** | **Indirectness** | **Imprecision** | **Publication bias** | **Intervention-1** | **Intervention-2** | **OR with 95% CI** |  |
| **PPV vs SB** [3, 4, 5, 9, 15, 16, 19] | | | | | | | | | | |
| **3** | RCT | Serious | Not serious | Not serious | Not serious | Undetected | 634 | 663 | 1.56 [0.91; 2.70] | Moderate |
| **PPV vs PPV+SB** [7, 11, 13, 14] | | | | | | | | | | |
| **2** | RCT | **Serious** | Not serious | Not serious | Not serious | Undetected | 272 | 267 | 1.24 [0.36; 4.20] | Moderate |
| **PR vs SB** [1, 2] | | | | | | | | | | |
| **2** | RCT | Serious | Not serious | Not serious | Serious | Undetected | 113 | 105 | 1.66 [0.31; 8.87] | Low |
| **PR vs PPV** [10] | | | | | | | | | | |
| **1** | RCT | Not Serious | Not Serious | Not serious | Not serious | Undetected | 77 | 73 | 0.94 [0.17; 5.25] | High |
| **PPV+SB vs PR** | | | | | | | | | | |
|  | RCT | Serious | Not serious | Not serious | Serious | Undetected |  |  | 0.76 [0.09; 6.29] | Moderate |
| **PPV+SB vs SB** | | | | | | | | | | |
|  | RCT | Serious | Not serious | Not serious | Not serious | Undetected |  |  | 1.27 [0.33; 4.84] | Low |

**The evidence findings for all comparisons (BCVA at 6 months)**

| **Certainty assessment** | | | | | | | **№ of Eyes** | | **Effect** | **Certainty** |
| --- | --- | --- | --- | --- | --- | --- | --- | --- | --- | --- |
| **№ of studies** | **Study design** | **Risk of bias** | **Inconsistency** | **Indirectness** | **Imprecision** | **Publication bias** | **Intervention-1** | **Intervention-2** | **OR with 95% CI** |  |
| **PPV vs SB** [5, 9, 12, 16, 19] | | | | | | | | | | |
| **5** | RCT | Serious | Not serious | Not serious | Not serious | Undetected | 233 | 266 | 0.08 [-0.02; 0.18] | Moderate |
| **PPV vs PPV+SB** [6, 11, 12, 13] | | | | | | | | | | |
| **4** | RCT | **Serious** | Not serious | Not serious | Not serious | Undetected | 179 | 203 | -0.06 [-0.16; 0.04] | Moderate |
| **PPV vs PCV** [8] | | | | | | | | | | |
| **1** | RCT | Serious | Not serious | Not serious | Not serious | Undetected | 29 | 30 | 0.09 [-0.11; 0.29] | Moderate |
| **PPV+SB vs SB** [12] | | | | | | | | | | |
| **1** | RCT | Serious | Not Serious | Not serious | Not serious | Undetected | 58 | 43 | 0.14 [ 0.01; 0.27] | Moderate |
| **PPV+SB vs PCV** | | | | | | | | | | |
|  | RCT | Serious | Not serious | Not serious | Not serious | Undetected |  |  | 0.03 [-0.19; 0.26] | Moderate |
| **SB vs PCV** | | | | | | | | | | |
|  | RCT | Serious | Not serious | Not serious | Not serious | Undetected |  |  | 0.17 [-0.05; 0.39] | Moderate |

**The evidence findings for all comparisons (Postoperative cataract progression)**

| **Certainty assessment** | | | | | | | **№ of Eyes** | | **Effect** | **Certainty** |
| --- | --- | --- | --- | --- | --- | --- | --- | --- | --- | --- |
| **№ of studies** | **Study design** | **Risk of bias** | **Inconsistency** | **Indirectness** | **Imprecision** | **Publication bias** | **Intervention-1** | **Intervention-2** | **OR with 95% CI** |  |
| **SB vs PPV** [3, 9, 19] | | | | | | | | | | |
| **3** | RCT | Serious | Not serious | Not serious | Serious | Undetected | 281 | 280 | 5.27 [3.13; 8.86] | Low |
| **PPV vs PPV+SB** [7] | | | | | | | | | | |
| **1** | RCT | **Serious** | Not serious | Not serious | Not serious | Undetected | 100 | 100 | 0.52 [0.26; 1.04] | Moderate |
| **PR vs PPV** [10] | | | | | | | | | | |
| **1** | RCT | Not serious | Not serious | Not serious | Very serious | Undetected | 77 | 73 | 7.51 [3.33; 16.91] | Low |
| **PPV+SB vs SB** [17] | | | | | | | | | | |
| **1** | RCT | Serious | Not Serious | Not serious | Very serious | Undetected | 4 | 5 | 10.12 [4.31; 23.77] | Very Low |
| **PR vs SB** [1] | | | | | | | | | | |
| **1** | RCT | Serious | Not serious | Not Serious | Not serious | Undetected | 57 | 51 | 0.70 [0.27; 1.80] | Moderate |
| **PPV+SB vs PR** | | | | | | | | | | |
|  | RCT | Serious | Not serious | Not serious | Very serious | Undetected |  |  | 14.43 [4.97; 41.93] | Very  Low |

**The evidence findings for all comparisons (Macular pucker)**

| **Certainty assessment** | | | | | | | **№ of Eyes** | | **Effect** | **Certainty** |
| --- | --- | --- | --- | --- | --- | --- | --- | --- | --- | --- |
| **№ of studies** | **Study design** | **Risk of bias** | **Inconsistency** | **Indirectness** | **Imprecision** | **Publication bias** | **Intervention-1** | **Intervention-2** | **OR with 95% CI** |  |
| **PPV vs SB** [4, 5, 9, 12, 15, 16, 19] | | | | | | | | | | |
| **7** | RCT | Serious | Not serious | Not serious | Not serious | Undetected | 331 | 364 | 0.85 [0.52; 1.37] | Moderate |
| **PPV vs PPV+SB** [12, 13] | | | | | | | | | | |
| **2** | RCT | **Serious** | Not serious | Not serious | Serious | Undetected | 66 | 88 | 1.44 [0.42; 4.89] | Low |
| **PPV+SB vs SB** [12,17] | | | | | | | | | | |
| **2** | RCT | Serious | Not serious | Not serious | Not  serious | Undetected | 20 | 20 | 0.59 [0.17; 2.00] | Moderate |
| **PR vs PPV** [10] | | | | | | | | | | |
| **1** | RCT | Not  serious | Not Serious | Not serious | Serious | Undetected | 77 | 73 | 1.14 [0.32; 4.08] | Moderate |
| **PR vs SB** [1] | | | | | | | | | | |
| **1** | RCT | Serious | Not serious | Not serious | Serious | Undetected | 103 | 95 | 0.74 [0.22; 2.50] | Low |
| **PPV+SB vs PR** | | | | | | | | | | |
|  | RCT | Serious | Not serious | Not serious | Serious | Undetected |  |  | 0.79 [0.14; 4.39] | Low |

**The evidence findings for all comparisons (Macular edema)**

| **Certainty assessment** | | | | | | | **№ of Eyes** | | **Effect** | **Certainty** |
| --- | --- | --- | --- | --- | --- | --- | --- | --- | --- | --- |
| **№ of studies** | **Study design** | **Risk of bias** | **Inconsistency** | **Indirectness** | **Imprecision** | **Publication bias** | **Intervention-1** | **Intervention-2** | **OR with 95% CI** |  |
| **PPV vs PPV+SB** [7, 12, 13] | | | | | | | | | | |
| **3** | RCT | Serious | Not serious | Not serious | Not serious | Undetected | 166 | 188 | 1.16 [0.36; 3.77] | Moderate |
| **PPV vs SB** [5, 12, 16] | | | | | | | | | | |
| **3** | RCT | **Serious** | Not serious | Not serious | Not serious | Undetected | 160 | 194 | 0.86 [0.26; 2.86] | Moderate |
| **PPV+SB vs SB** [12, 17] | | | | | | | | | | |
| **2** | RCT | Serious | Not serious | Not serious | Not serious | Undetected | 78 | 63 | 0.74 [0.17; 3.14] | Moderate |
| **PPV vs PCV** [8] | | | | | | | | | | |
| **1** | RCT | Serious | Not Serious | Not serious | Not serious | Undetected | 29 | 30 | 15.65 [0.56; 435.46] | Moderate |
| **PPV vs PR** [10] | | | | | | | | | | |
| **1** | RCT | Not serious | Not serious | Not serious | Serious | Undetected | 73 | 77 | 1.77 [0.27; 11.57] | Moderate |
| **PPV+SB vs PCV** | | | | | | | | | | |
|  | RCT | Serious | Not serious | Serious | Very serious | Undetected |  |  | 18.18 [0.53; 618.82] | Very  Low |
| **PR vs PCV** | | | | | | | | | | |
|  | RCT | Serious | Not serious | Not serious | Very serious | Undetected |  |  | 27.77 [0.61; 1263.62] | Very  Low |
| **SB vs PCV** | | | | | | | | | | |
|  | RCT | Serious | Not Serious | Not serious | Very serious | Undetected |  |  | 13.47 [0.39; 462.29] | Very Low |
| **PPV+SB vs PR** | | | | | | | | | | |
|  | RCT | Serious | Not serious | Not serious | Serious | Undetected |  |  | 1.53 [0.17; 13.97] | Low |
| **PR vs SB** | | | | | | | | | | |
|  | RCT | Serious | Not serious | Not serious | Not serious | Undetected |  |  | 0.48 [0.05; 4.49] | Moderate |

**The evidence findings for all comparisons (Missed/new breaks)**

| **Certainty assessment** | | | | | | | **№ of Eyes** | | **Effect** | **Certainty** |
| --- | --- | --- | --- | --- | --- | --- | --- | --- | --- | --- |
| **№ of studies** | **Study design** | **Risk of bias** | **Inconsistency** | **Indirectness** | **Imprecision** | **Publication bias** | **Intervention-1** | **Intervention-2** | **OR with 95% CI** |  |
| **SB vs PPV** [4, 5, 9, 16, 19] | | | | | | | | | | |
| **5** | RCT | Serious | Not serious | Not serious | Not serious | Undetected | 298 | 272 | 0.77 [0.36; 1.65] | Moderate |
| **PR vs SB** [1, 2] | | | | | | | | | | |
| **2** | RCT | **Serious** | Not serious | Not serious | Serious | Undetected | 113 | 105 | 2.01 [0.96; 4.18] | Low |
| **PPV+SB vs PPV** [14] | | | | | | | | | | |
| **1** | RCT | Serious | Not serious | Not serious | Serious | Undetected | 38 | 44 | 1.05 [0.31; 3.51] | Low |
| **PPV+SB vs SB** [17] | | | | | | | | | | |
| **1** | RCT | Serious | Not Serious | Not serious | Serious | Undetected | 20 | 20 | 0.73 [0.20; 2.73] | Low |
| **PR vs PPV** | | | | | | | | | | |
|  | RCT | Not serious | Not serious | Not serious | Not serious | Undetected |  |  | 0.38 [0.13; 1.11] | High |
| **PPV+SB vs PR** | | | | | | | | | | |
|  | RCT | **Serious** | Not serious | Not serious | Not serious | Undetected |  |  | 0.37 [0.08; 1.65] | Moderate |

**The evidence findings for all comparisons (PVR)**

| **Certainty assessment** | | | | | | | **№ of Eyes** | | **Effect** | **Certainty** |
| --- | --- | --- | --- | --- | --- | --- | --- | --- | --- | --- |
| **№ of studies** | **Study design** | **Risk of bias** | **Inconsistency** | **Indirectness** | **Imprecision** | **Publication bias** | **Intervention-1** | **Intervention-2** | **OR with 95% CI** |  |
| **SB vs PPV** [3, 4, 5, 15, 16, 19] | | | | | | | | | | |
| **6** | RCT | Serious | Not serious | Not serious | Not serious | Undetected | 622 | 591 | 1.02 [0.75; 1.39] | Moderate |
| **PPV+SB vs PPV** [7, 11, 13, 14] | | | | | | | | | | |
| **4** | RCT | **Serious** | Not serious | Not serious | Not serious | Undetected | 265 | 270 | 0.85 [0.47; 1.51] | Moderate |
| **PR vs SB** [1, 2] | | | | | | | | | | |
| **2** | RCT | Serious | Not serious | Not serious | Serious | Undetected | 113 | 105 | 0.83 [0.22; 3.11] | Low |
| **PPV+SB vs SB** [17] | | | | | | | | | | |
| **1** | RCT | Serious | Not Serious | Not serious | Not serious | Undetected | 20 | 20 | 1.21 [0.64; 2.28] | Moderate |
| **PR vs PPV** [ | | | | | | | | | | |
|  | RCT | Not serious | Not serious | Not serious | Serious | Undetected |  |  | 1.23 [0.32; 4.81] | Low |
| **PPV+SB vs PR** | | | | | | | | | | |
|  | RCT | **Serious** | Not serious | Not serious | Serious | Undetected |  |  | 1.46 [0.33; 6.35] | Low |

The results pertaining to rows with 0 number of studies and no cited articles, are derived through the process of indirect comparison in network meta-analysis.

**Reference:**

**1.** Tornambe, P. E., Hilton, G. F., Poliner, L. S., Brinton, D. A., Flood, T. P., Orth, D. H., ... & Tiedeman, J. S. (1989). Pneumatic retinopexy: a multicenter randomized controlled clinical trial comparing pneumatic retinopexy with scleral buckling. *Ophthalmology*, *96*(6), 772-784.

**2.** Mulvihill, A., Fulcher, T., Datta, V., & Acheson, R. (1996). Pneumatic retinopexy versus scleral buckling: a randomised controlled trial. *Irish Journal of medical science*, *165*, 274-277.

3. Heimann, H., Bartz-Schmidt, K. U., Bornfeld, N., Weiss, C., Hilgers, R. D., Foerster, M. H., & Scleral Buckling versus Primary Vitrectomy in Rhegmatogenous Retinal Detachment Study Group. (2007). Scleral buckling versus primary vitrectomy in rhegmatogenous retinal detachment: a prospective randomized multicenter clinical study. *Ophthalmology*, *114*(12), 2142-2154.

4. Brazitikos, P. D., Androudi, S., Christen, W. G., & Stangos, N. T. (2005). Primary pars plana vitrectomy versus scleral buckle surgery for the treatment of pseudophakic retinal detachment: a randomized clinical trial. *Retina*, *25*(8), 957-964.

5. Sharma, Y. R., Karunanithi, S., Azad, R. V., Vohra, R., Pal, N., Singh, D. V., & Chandra, P. (2005). Functional and anatomic outcome of scleral buckling versus primary vitrectomy in pseudophakic retinal detachment. *Acta Ophthalmologica Scandinavica*, *83*(3), 293-297.

6. KHAN, R., KHAN, A. A., & CH, N. A. (2019). Scleral buckling and pars plana vitrectomy versus pars plana vitrectomy alone in the treatment of rhegmatogenous retinal detachment with inferior break. *Age (years)*, *42*, 12-5.

7. Mehboob, M. A. R. I. A., Ghani, M. U., Khan, A. S. M. A., & Imran, M. (2018). Scleral buckling and pars plana vitrectomy versus vitrectomy alone for primary repair of rhegmatogenous retinal detachment. *Pakistan J Med Health Sci*, *12*, 716-720.

8. Mora, P., Favilla, S., Calzetti, G., Berselli, G., Benatti, L., Carta, A., ... & Tedesco, S. A. (2021). Parsplana vitrectomy alone versus parsplana vitrectomy combined with phacoemulsification for the treatment of rhegmatogenous retinal detachment: a randomized study. *BMC ophthalmology*, *21*, 1-7.

9. Zhao, X., Huang, L., Lyu, C., Liu, B., Ma, W., Deng, X., ... & Lu, L. (2020). Comparison between releasable scleral buckling and vitrectomy in patients with phakic primary rhegmatogenous retinal detachment. *Retina (Philadelphia, Pa.)*, *40*(1), 33.

10. Hillier, R. J., Felfeli, T., Berger, A. R., Wong, D. T., Altomare, F., Dai, D., ... & Muni, R. H. (2019). The pneumatic retinopexy versus vitrectomy for the management of primary rhegmatogenous retinal detachment outcomes randomized trial (PIVOT). *Ophthalmology*, *126*(4), 531-539.

11. Walter, P., Hellmich, M., Baumgarten, S., Schiller, P., Limburg, E., Agostini, H., ... & Mazinani, B. (2017). Vitrectomy with and without encircling band for pseudophakic retinal detachment: VIPER Study Report No 2—main results. *British Journal of Ophthalmology*, *101*(6), 712-718.

12. Moradian, S., Ahmadieh, H., Faghihi, H., Ramezani, A., Entezari, M., Banaee, T., ... & Yasseri, M. (2016). Comparison of four surgical techniques for management of pseudophakic and aphakic retinal detachment: a multicenter clinical trial. *Graefe's Archive for Clinical and Experimental Ophthalmology*, *254*, 1743-1751.

13. Falkner‐Radler, C. I., Graf, A., & Binder, S. (2015). Vitrectomy combined with endolaser or an encircling scleral buckle in primary retinal detachment surgery: a pilot study. *Acta ophthalmologica*, *93*(5), 464-469.

14. Romano, M. R., Angi, M., Valldeperas, X., Costagliola, C., & Vinciguerra, P. (2011). Twenty-three–gauge pars plana vitrectomy, densiron-68, and 360 endolaser versus combined 20-gauge pars plana vitrectomy, scleral buckle, and SF6 for pseudophakic retinal detachment with inferior retinal breaks. *Retina*, *31*(4), 686-691.

15. Koriyama, M., Nishimura, T., Matsubara, T., Taomoto, M., Takahashi, K., & Matsumura, M. (2007). Prospective study comparing the effectiveness of scleral buckling to vitreous surgery for rhegmatogenous retinal detachment. *Japanese journal of ophthalmology*, *51*, 360-367.

16. Ahmadieh H, Moradian S, Faghihi H, et al. Anatomic and visual outcomes of scleral buckling versus primary vitrectomy in pseudophakic and aphakic retinal detachment: six-month follow-up results of a single operation--report no. 1. *Ophthalmology*. 2005;112(8):1421-1429. doi:10.1016/j.ophtha.2005.02.018

17. Tewari, H. K., Kedar, S., Kumar, A., Garg, S. P., & Verma, L. K. (2003). Comparison of scleral buckling with combined scleral buckling and pars plana vitrectomy in the management of rhegmatogenous retinal detachment with unseen retinal breaks: Clinical Research. *Clinical & experimental ophthalmology*, *31*(5), 403-407.

18. Dahab, A. A., Helmy, Y. A., Khattab, A. M., Abdelhakim, M. A., & Hamza, H. S. (2020). Vitrectomy and silicone oil tamponade with and without phacoemulsification in the management of rhegmatogenous retinal detachment: A comparative study. *African Vision and Eye Health*, *79*(1), 1-8.

19. Azad, R. V., Chanana, B., Sharma, Y. R., & Vohra, R. (2007). Primary vitrectomy versus conventional retinal detachment surgery in phakic rhegmatogenous retinal detachment. *Acta Ophthalmologica Scandinavica*, *85*(5), 540-545.
