# Supplementary material for: Surgical managements for rhegmatogenous retinal detachment: A network meta-analysis of randomized controlled trial
Source: PLoS One. 2024 Nov 14;19(11):e0310859. doi: 10.1371/journal.pone.0310859 (PMC11563380; doi:10.1371/journal.pone.0310859)
Supplement: S7 File — (DOCX) [file pone.0310859.s007.docx]

**S7 File. SUCRA values (%) for all outcomes.**

**SUCRA values (%) for primary outcomes**

| **Intervention** | **Primary Reattachment Rate** | **Final Reattachment Rate** | **BCVA at 6months** |
| --- | --- | --- | --- |
| PPV | 59% | 71% | 37% |
| SB | 29% | 27% | 6% |
| PR | 1% | 51% | -- |
| PCV | 93% | -- | 81% |
| PPV+SB | 68% | 51% | 77% |

**SUCRA value (%) for secondary outcomes**

| **Intervention** | **Mcaular edema** | **Macular pucker** | **Miss/new breaks** | **Cataract Progression** | **PVR** |
| --- | --- | --- | --- | --- | --- |
| PPV | 47% | 53% | 20% | 35% | 50% |
| SB | 52% | 75% | 56% | 61% | 43% |
| PR | 23% | 49% | 95% | 39% | 39% |
| PCV | 96% | -- | -- | -- | -- |
| PPV+SB | 32% | 23% | 29% | 65% | 68% |
