# Supplementary material for: Surgical managements for rhegmatogenous retinal detachment: A network meta-analysis of randomized controlled trial
Source: PLoS One. 2024 Nov 14;19(11):e0310859. doi: 10.1371/journal.pone.0310859 (PMC11563380; doi:10.1371/journal.pone.0310859)
Supplement: S8 File — (DOCX) [file pone.0310859.s008.docx]

**S8 File. Node-splitting test for inconsistency.**

**Node-splitting test for inconsistency (Primary retinal reattachment rate)**

| **Intervention** | **Control** | **Network** | **Direct** | **Indirect** | **P value** |
| --- | --- | --- | --- | --- | --- |
| PPV | SB | 1.22 [0.97; 1.55] | 1.21 [0.95; 1.54] | 1.42 [0.59; 3.42] | 0.722 |
| PPV | PPV+SB | 0.91 [0.63; 1.32] | 0.85 [0.57; 1.26] | 1.49 [0.52; 4.23] | 0.324 |
| PPV | PCV | 0.30 [0.05; 2.02] | 0.30 [0.05; 2.02] | -- | -- |
| PPV+SB | SB | 1.34 [0.89; 2.03] | 0.95 [0.45; 2.02] | 1.56 [0.95; 2.56] | 0.281 |
| PR | SB | 0.52 [0.30; 0.91] | 0.58 [0.31; 1.11] | 0.37 [0.12; 1.11] | 0.486 |
| PPV | PR | 2.35 [1.32; 4.20] | 3.24 [1.11; 9.43] | 2.06 [1.04; 4.11] | 0.486 |
| PPV+SB | PCV | 0.33 [0.05; 2.30] | -- | 0.33 [0.05; 2.30] | -- |
| PR | PCV | 0.13 [0.02; 0.94] | -- | 0.13 [0.02; 0.94] | -- |
| SB | PCV | 0.25 [0.04; 1.68] | -- | 0.25 [0.04; 1.68] | -- |
| PPV+SB | PR | 2.59 [1.32; 5.09] | -- | 2.59 [1.32; 5.09] | -- |

**Node-splitting test for inconsistency (Final retinal reattachment rate)**

| **Intervention** | **Control** | **Network** | **Direct** | **Indirect** | **P value** |
| --- | --- | --- | --- | --- | --- |
| SB | PPV | 0.64 [0.37; 1.10] | 0.62 [0.36; 1.07] | 2.91 [0.07; 125.32] | 0.425 |
| PPV+SB | PPV | 0.81 [0.24; 2.75] | 0.81 [0.24; 2.75] | -- | -- |
| PR | SB | 1.66 [0.31; 8.87] | 1.10 [0.16; 7.78] | 5.18 [0.20; 135.41] | 0.425 |
| PR | PPV | 1.06 [0.19; 5.90] | 3.21 [0.13; 79.99] | 0.68 [0.09; 5.20] | 0.425 |
| PPV+SB | PR | 0.76 [0.09; 6.29] | -- | 0.76 [0.09; 6.29] | -- |
| PPV+SB | SB | 1.27 [0.33; 4.84] | -- | 1.27 [0.33; 4.84] | -- |

**Node-splitting test for inconsistency (BCVA at 6 months)**

| **Intervention** | **Control** | **Network** | **Direct** | **Indirect** | **P value** |
| --- | --- | --- | --- | --- | --- |
| PPV | SB | 0.08 [-0.02; 0.18] | 0.10 [ 0.00; 0.20] | -0.25 [-0.67; 0.17] | 0.113 |
| PPV | PPV+SB | -0.06 [-0.16; 0.04] | -0.07 [-0.17; 0.03] | 0.12 [-0.28; 0.52] | 0.373 |
| PPV | PCV | -0.09 [-0.29; 0.11] | -0.09 [-0.29; 0.11] | -- | -- |
| PPV+SB | SB | 0.14 [ 0.01; 0.27] | 0.02 [-0.18; 0.22] | 0.22 [ 0.05; 0.38] | 0.141 |
| PPV+SB | PCV | -0.03 [-0.26; 0.19] | -- | -0.03 [-0.26; 0.19] | -- |
| SB | PCV | -0.17 [-0.39; 0.05] | -- | -0.17 [-0.39; 0.05] | -- |

**Node-splitting test for inconsistency (Macular pucker)**

| **Intervention** | **Control** | **Network** | **Direct** | **Indirect** | **P value** |
| --- | --- | --- | --- | --- | --- |
| SB | PPV | 1.18 [0.73; 1.91] | 1.15 [0.70; 1.87] | 2.40 [0.23; 25.25] | 0.548 |
| PPV+SB | PPV | 0.69 [0.20; 2.36] | 0.69 [0.17; 2.79] | 0.72 [0.06; 8.89] | 0.977 |
| PPV+SB | SB | 0.59 [0.17; 2.00] | 0.32 [0.08; 1.39] | 2.44 [0.26; 23.05] | 0.139 |
| PR | PPV | 0.87 [0.24; 3.12] | 0.95 [0.06; 15.43] | 0.86 [0.20; 3.58] | 0.95 |
| PR | SB | 0.74 [0.22; 2.50] | 0.73 [0.19; 2.79] | 0.80 [0.05; 13.66] | 0.95 |
| PPV+SB | PR | 0.79 [0.14; 4.39] | -- | 0.79 [0.14; 4.39] | -- |

**Node-splitting test for inconsistency (Macular edema)**

| **Intervention** | **Control** | **Network** | **Direct** | **Indirect** | **P value** |
| --- | --- | --- | --- | --- | --- |
| PPV | PPV+SB | 1.16 [0.36; 3.77] | 1.00 [0.29; 3.53] | 3.19 [0.11; 88.68] | 0.524 |
| PPV | SB | 0.86 [0.26; 2.86] | 1.16 [0.31; 4.30] | 0.17 [0.01; 3.56] | 0.257 |
| PPV+SB | SB | 0.74 [0.17; 3.14] | 0.27 [0.04; 2.03] | 2.18 [0.27; 17.52] | 0.16 |
| PPV | PCV | 0.06 [0.00; 1.78] | 0.06 [0.00; 1.78] | -- | -- |
| PPV | PR | 1.77 [0.27; 11.57] | 1.77 [0.27; 11.57] | -- | -- |
| PPV+SB | PCV | 0.06 [0.00; 1.87] | -- | 0.06 [0.00; 1.87] | -- |
| PR | PCV | 0.04 [0.00; 1.64] | -- | 0.04 [0.00; 1.64] | -- |
| SB | PCV | 0.07 [0.00; 2.55] | -- | 0.07 [0.00; 2.55] | -- |
| PPV+SB | PR | 1.53 [0.17;13.97] | -- | 1.53 [0.17;13.97] | -- |
| PR | SB | 0.48 [0.05; 4.49] | -- | 0.48 [0.05; 4.49] | -- |

**Node-splitting test for inconsistency (Missed/new breaks)**

| **Intervention** | **Control** | **Network** | **Direct** | **Indirect** | **P value** |
| --- | --- | --- | --- | --- | --- |
| SB | PPV | 1.30 [0.61; 2.79] | 1.12 [0.50; 2.48] | 7.20 [0.49; 105.02] | 0.192 |
| PR | SB | 2.01 [0.96; 4.18] | 2.01 [0.96; 4.18] | -- | -- |
| PPV+SB | PPV | 0.95 [0.29; 3.19] | 1.52 [0.38; 6.10] | 0.24 [0.02; 2.66] | 0.192 |
| PPV+SB | SB | 0.73 [0.20; 2.73] | 0.21 [0.02; 2.08] | 1.36 [0.27; 6.75] | 0.192 |
| PR | PPV | 2.60 [0.90; 7.51] | -- | 2.60 [0.90; 7.51] | -- |
| PPV+SB | PR | 0.37 [0.08; 1.65] | -- | 0.37 [0.08; 1.65] | -- |

**Node-splitting test for inconsistency (PVR)**

| **Intervention** | **Control** | **Network** | **Direct** | **Indirect** | **P value** |
| --- | --- | --- | --- | --- | --- |
| SB | PPV | 0.98 [0.72; 1.34] | 0.95 [0.69; 1.30] | 2.53 [0.46; 13.97] | 0.268 |
| PPV+SB | PPV | 1.18 [0.66; 2.11] | 1.34 [0.72; 2.50] | 0.50 [0.10; 2.54] | 0.268 |
| PR | SB | 0.83 [0.22; 3.11] | 0.83 [0.22; 3.11] | -- | -- |
| PPV+SB | SB | 1.21 [0.64; 2.28] | 0.53 [0.11; 2.60] | 1.41 [0.70; 2.84] | 0.268 |
| PR | PPV | 0.81 [0.21; 3.16] | -- | 0.81 [0.21; 3.16] | -- |
| PPV+SB | PR | 1.46 [0.33; 6.35] | -- | 1.46 [0.33; 6.35] | -- |

**Node-splitting test for inconsistency (Cataract progression)**

| **Intervention** | **Control** | **Network** | **Direct** | **Indirect** | **P value** |
| --- | --- | --- | --- | --- | --- |
| SB | PPV | 0.19 [0.11; 0.32] | 0.19 [0.11; 0.33] | 0.16 [0.02; 1.50] | 0.866 |
| PPV+SB | PPV | 1.92 [0.96; 3.84] | 1.92 [0.94; 3.88] | 2.09 [0.07; 66.04] | 0.961 |
| PR | PPV | 0.13 [0.06; 0.30] | 0.13 [0.06; 0.30] | 0.17 [0.01; 3.04] | 0.859 |
| PPV+SB | SB | 10.12 [4.31; 23.77] | 11.00 [0.36; 333.34] | 10.07 [4.17; 24.31] | 0.961 |
| PR | SB | 0.70 [0.27; 1.80] | 0.89 [0.05; 15.13] | 0.68 [0.25; 1.85] | 0.859 |
| PPV+SB | PR | 14.43 [4.97; 41.93] | -- | 14.43 [4.97; 41.93] | -- |
