# Supplementary material for: Surgical managements for rhegmatogenous retinal detachment: A network meta-analysis of randomized controlled trial
Source: PLoS One. 2024 Nov 14;19(11):e0310859. doi: 10.1371/journal.pone.0310859 (PMC11563380; doi:10.1371/journal.pone.0310859)
Supplement: S9 File — (DOCX) [file pone.0310859.s009.docx]

**S9 File. Network meta-regression results for abstinence rate**

| **Outcomes** | **Covariates** | **Beta** | **50%** | **95%Crl** |
| --- | --- | --- | --- | --- |
| Primary retinal reattachment rate | Lens status (Phakic) | B | 0.23 | (-0.57, 0.94) |
|  | PVR Grade (Baseline) | B | 0.23 | (-1.49, 2.11) |
|  | RRD severity | B | 0.17 | (-7.51, 25.30) |
|  | Age (years) | B | 0.40 | (-0.27, 1.06) |
|  | Publication year | B | 0.48 | (-0.38, 1.36) |
|  | ROB | B | 0.16 | (-0.40, 0.92) |
|  | Sample size (eyes) | B | 0.07 | (-0.40, 0.47) |
| Final retinal reattachment rate | Lens status (Phakic） | B | -0.52 | (-2.48, 1.04) |
|  | PVR Grade (Baseline) | B | 0.09 | (-11.47, 131.40) |
|  | RRD severity | B | 0.02 | (-21.90, 29.64) |
|  | Age (years) | B | 1.38 | (-0.90, 6.10) |
|  | Publication year | B | -0.01 | (-7.44, 9.88) |
|  | ROB | B | 0.32 | (-1.02, 1.71) |
|  | Sample size (eyes) | B | -0.18 | (-1.20, 0.94) |
| BCVA at 6 months | Lens status (Phakic) | B | 0.47 | (-1.25, 2.36) |
|  | PVR Grade (Baseline) | B | 2.54 | (-0.84, 19.99) |
|  | RRD severity | B | -0.01 | (-9.83, 8.94) |
|  | Age (years) | B | -0.17 | (-2.21, 1.50) |
|  | Publication year | B | 0.02 | (-1.94, 1.77) |
|  | ROB | B | 0.53 | (-1.29, 2.34) |
|  | Sample size (eyes) | B | 0.41 | (-1.26, 2.08) |
| Macular pucker | Lens status (Phakic) | B | 0.50 | (-1.18, 2.38) |
|  | PVR Grade (Baseline) | B | 2.75 | (-0.79, 60.85) |
|  | RRD severity | B | -0.04 | (-12.64, 11.14) |
|  | Age (years) | B | -0.16 | (-2.21, 1.50) |
|  | Publication year | B | 0.04 | (-1.94, 1.86) |
|  | ROB | B | 0.53 | (-1.28, 2.36) |
|  | Sample size (eyes) | B | 0.41 | (-1.26, 2.04) |
| Macular Edema | Lens status (Phakic) | B | -0.22 | (-9.91, 9.80) |
|  | PVR Grade (Baseline) | B | -0.30 | (-3.08, 2.38) |
|  | RRD severity | B | 0.04 | (-10.33, 11.81) |
|  | Age (years) | B | 0.83 | (-1.94, 3.52) |
|  | Publication year | B | 0.64 | (-2.46, 3.53) |
|  | ROB | B | 0.58 | (-2.18, 3.23) |
|  | Sample size (eyes) | B | 0.61 | (-2.17, 3.23) |
| Missed/new breaks | Lens status (Phakic) | B | -0.52 | (-2.40, 1.08) |
|  | PVR Grade (Baseline) | B | 0.23 | (-10.29, 9.46) |
|  | RRD severity | B | -0.04 | (-12.80, 13.03) |
|  | Age (years) | B | 1.42 | (-0.85, 7.19) |
|  | Publication year | B | 0.24 | (-8.42, 28.56) |
|  | ROB | B | 0.31 | (-1.08, 1.68) |
|  | Sample size (eyes) | B |  |  |
| PVR | Lens status (Phakic) | B | 0.75 | (-0.70, 3.36) |
|  | PVR Grade (Baseline) | B | -0.97 | (-4.72, 2.00) |
|  | RRD severity | B | 0.10 | (-15.63, 14.47) |
|  | Age (years) | B | -0.28 | (-2.24, 1.21) |
|  | Publication year | B | 0.27 | (-7.21, 23.43) |
|  | ROB | B | -0.43 | (-2.46, 0.79) |
|  | Sample size (eyes) | B | -0.08 | (-1.60, 1.34) |
| Cataract progression | Lens status (Phakic) | B | 0.76 | (-2.74, 3.76) |
|  | PVR Grade (Baseline) | B | 2.32 | (-2.04, 66.09) |
|  | Age (years) | B | -1.39 | (-4.41, 0.81) |
|  | Publication year | B | -0.57 | (-3.19,2.91) |
|  | ROB | B | 0.79 | (-2.29, 3.24) |
|  | Sample size (eyes) | B | -1.10 | (-3.27, 0.76) |
