# Supplementary material for: Surgical managements for rhegmatogenous retinal detachment: A network meta-analysis of randomized controlled trial
Source: PLoS One. 2024 Nov 14;19(11):e0310859. doi: 10.1371/journal.pone.0310859 (PMC11563380; doi:10.1371/journal.pone.0310859)
Supplement: S10 File — (DOCX) [file pone.0310859.s010.docx]

**S10 File. NMA results with corresponding certainty of evidence.**

**NMA results with corresponding certainty of evidence for postoperative cataract progression (upper triangle) and macular pucker (lower triangle).**

**
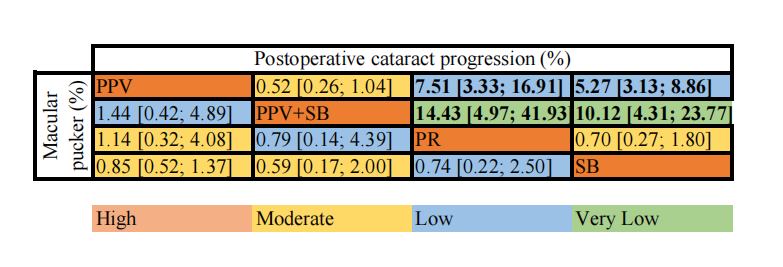
**

The above table shows the results of network meta-analysis of incidence of macular pucker and postoperative cataract progression, the corresponding GRADE (Recommendation, Evaluation, Development, and Evaluation Grade) evidence certainty. The values in the table represent OR values with 95% confidence intervals, each of which compares the two interventions. Values in bold indicate a statistically significant treatment effect.

**NMA results with corresponding certainty of evidence for PVR (lower triangle) and missed/new breaks (upper triangle).**

**
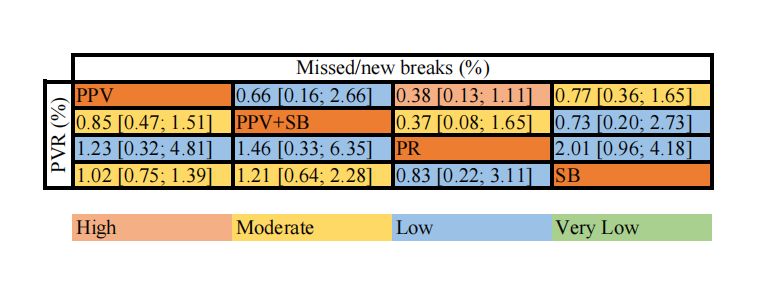
**

The above table shows the results of a network meta-analysis of the PVR and missed/new breaks and the corresponding GRADE (Recommendation, Evaluation, Development, and Evaluation Grade) evidence certainty. The values in the table represent OR values with 95% confidence intervals, each of which compares the two interventions. Values in bold indicate a statistically significant treatment effect.

**NMA results with corresponding certainty of evidence for macular edema.**

**
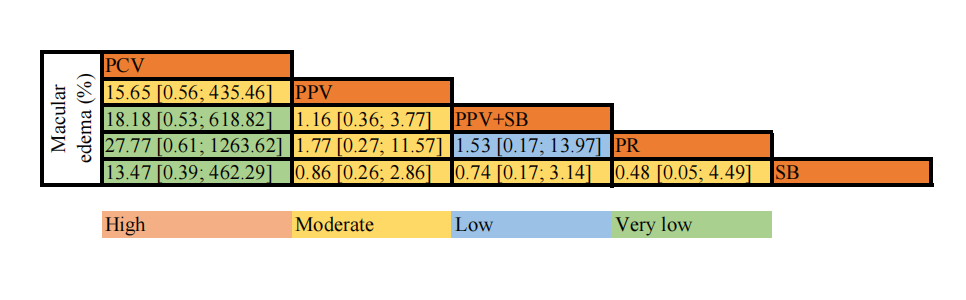
**

The above table shows the results of a network meta-analysis of incidence of postoperative macular edema and the corresponding GRADE (Recommendation, Evaluation, Development, and Evaluation Grade) evidence certainty. The values in the table represent OR values with 95% confidence intervals, each of which compares the two interventions. Values in bold indicate a statistically significant treatment effect.
