# Supplementary material for: Surgical managements for rhegmatogenous retinal detachment: A network meta-analysis of randomized controlled trial
Source: PLoS One. 2024 Nov 14;19(11):e0310859. doi: 10.1371/journal.pone.0310859 (PMC11563380; doi:10.1371/journal.pone.0310859)

**S11 File. Network Plots**

**Network plots for primary reattachment rate.**


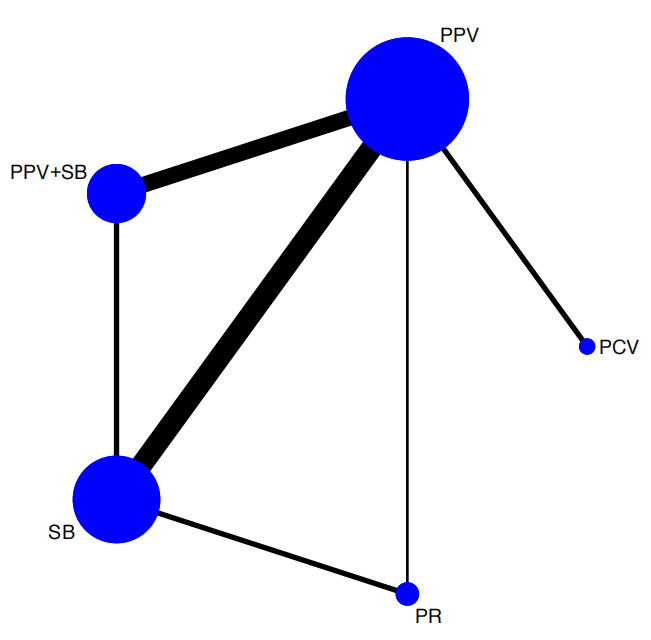


**Network plots for final reattachment rate.**

**
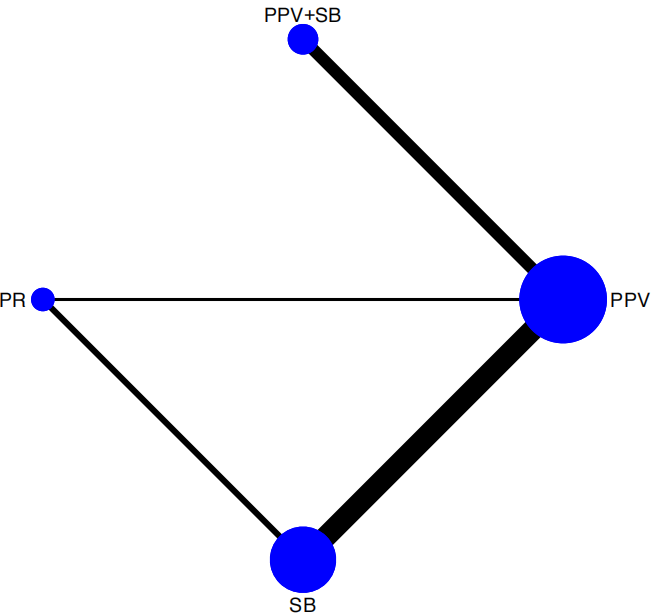
**

**Network plots for BCVA at 6 months.**

**
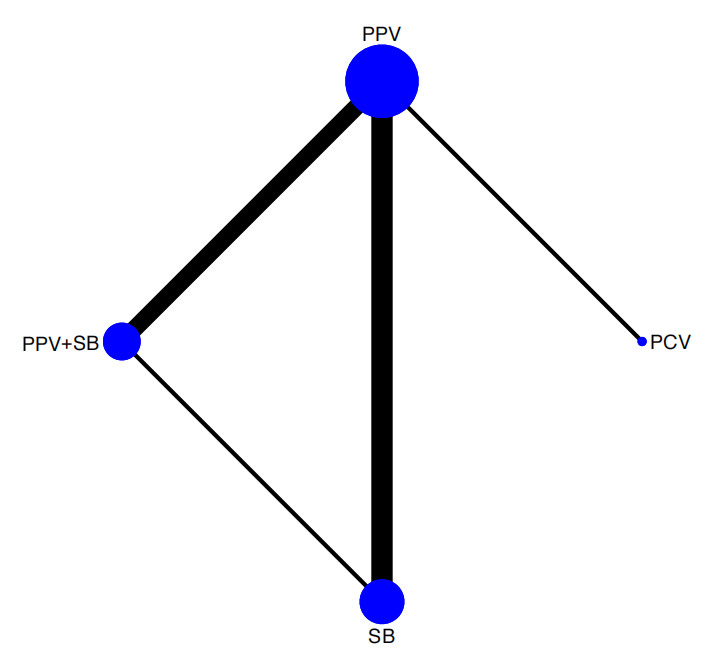
**

**Network plots for** **Cataract progression.**


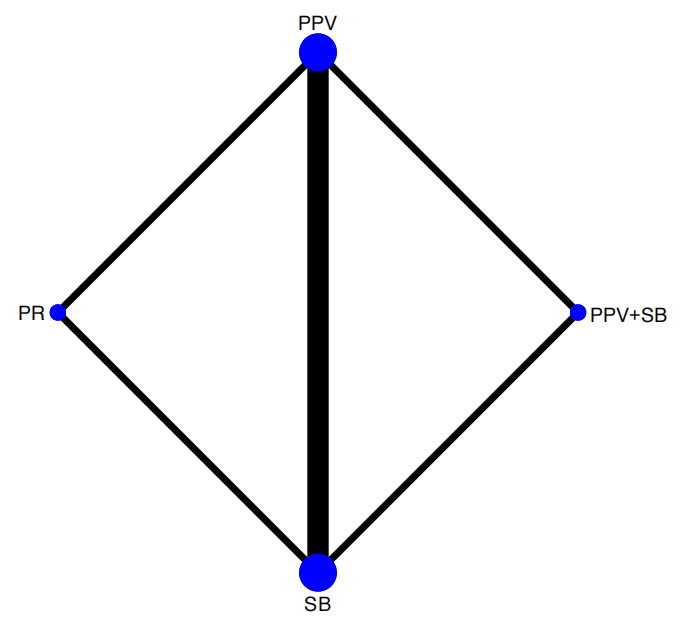


**Network plots for PVR.**

**
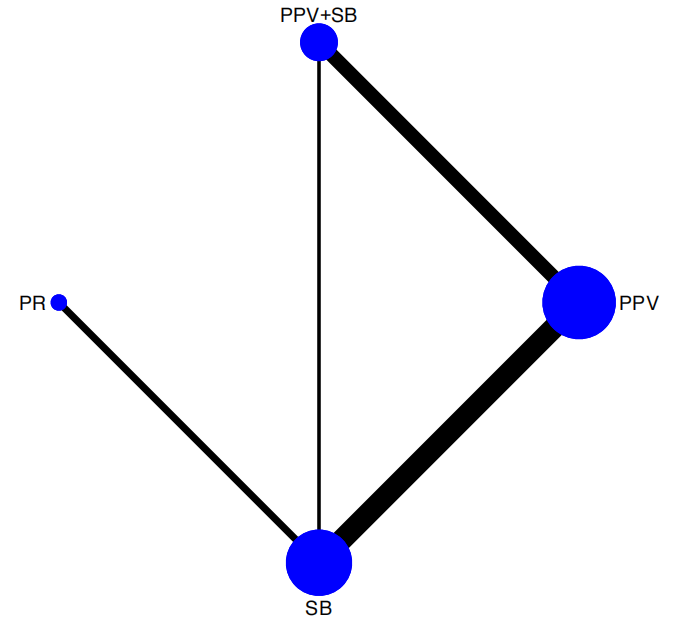
**

**Network plots for missed/new breaks**


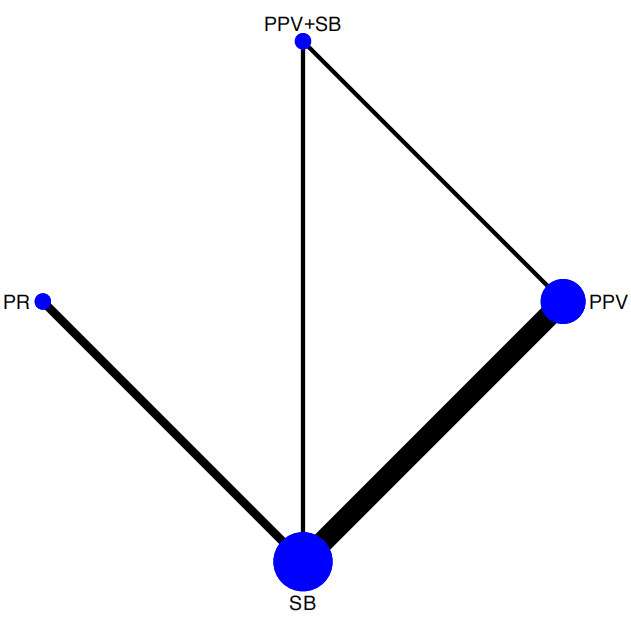


**Network plots for macular edema.**


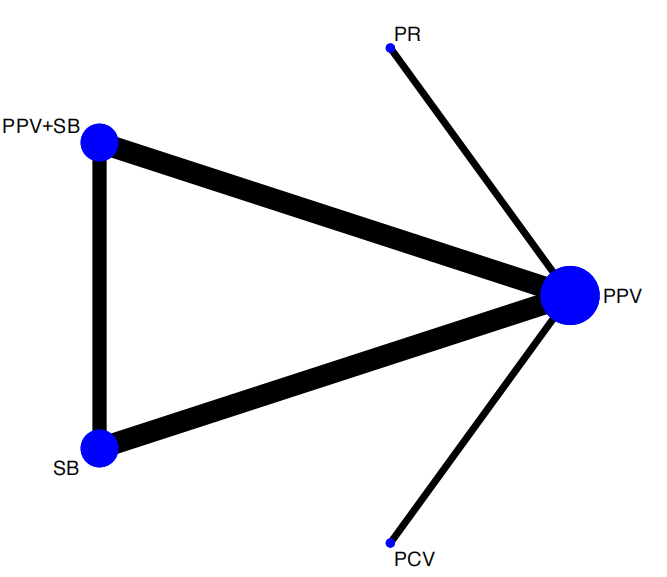


**Network plots for macular pucker.**


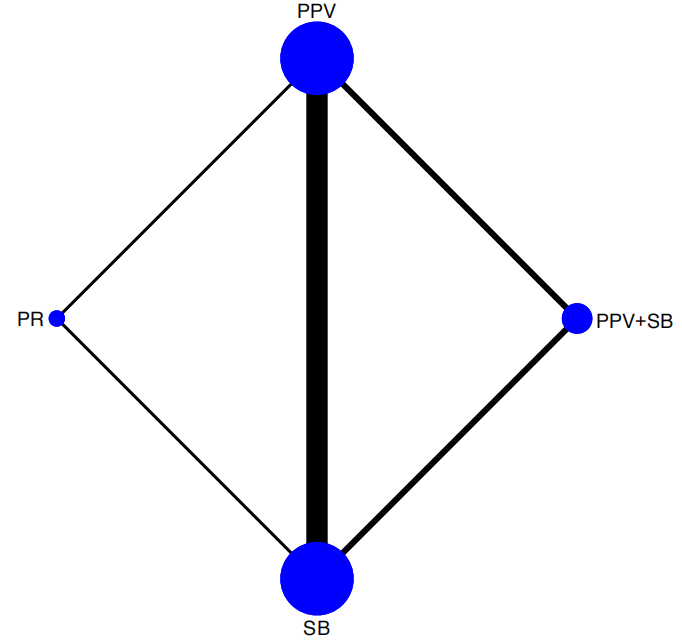

Supplement: S11 File — (DOCX) [file pone.0310859.s011.docx]
