# Supplementary figures and images for: Surgical managements for rhegmatogenous retinal detachment: A network meta-analysis of randomized controlled trial
Source: PLoS One. 2024 Nov 14;19(11):e0310859. doi: 10.1371/journal.pone.0310859 (PMC11563380; doi:10.1371/journal.pone.0310859)

**S12 File. Traffic light of risk of bias of included studies**


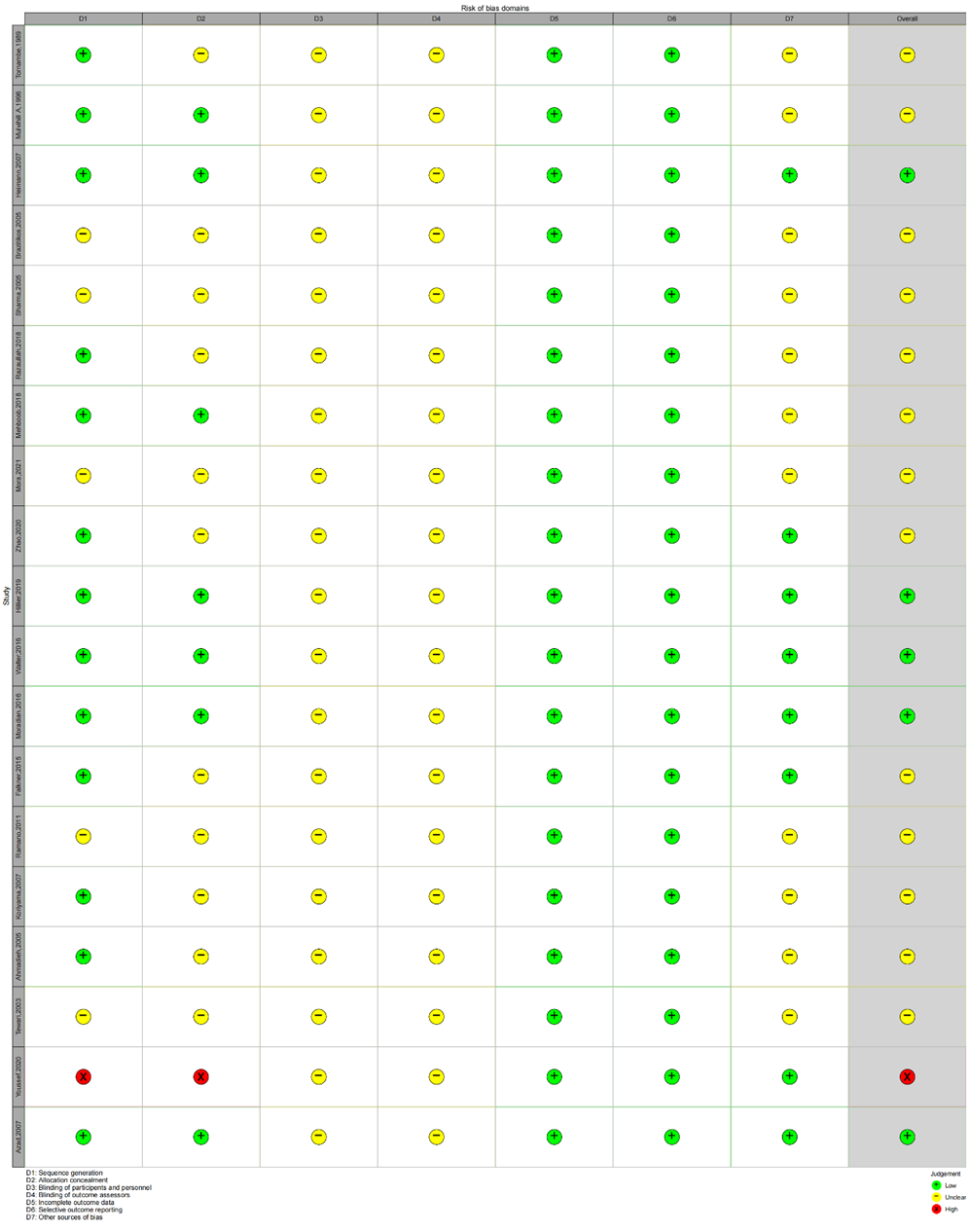

Supplement: S12 File — (DOCX) [file pone.0310859.s012.docx]

**S13 File: Summary graph of risk of bias of included studies**


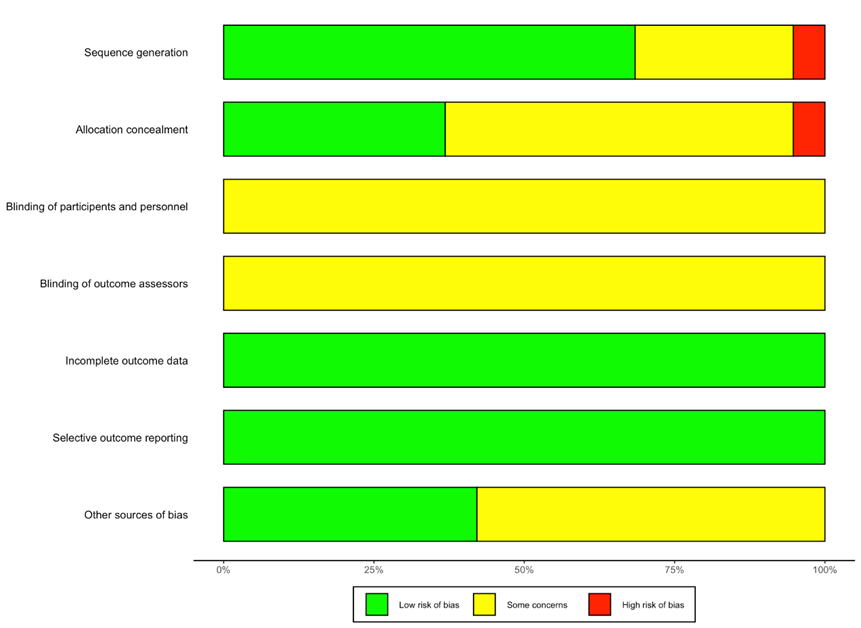

Supplement: S13 File — (DOCX) [file pone.0310859.s013.docx]
