# Supplementary material for: Surgical managements for rhegmatogenous retinal detachment: A network meta-analysis of randomized controlled trial
Source: PLoS One. 2024 Nov 14;19(11):e0310859. doi: 10.1371/journal.pone.0310859 (PMC11563380; doi:10.1371/journal.pone.0310859)
Supplement: S15 File — (DOCX) [file pone.0310859.s015.docx]

**S15 File: List of excluded studies during full-text screening and reasons for their exclusions**

| **ID** | **1st Author** | **year** | **Title** | **Reason for Exclusion** |
| --- | --- | --- | --- | --- |
| 1 | Roxane J Hillier | 2019 | The Pneumatic Retinopexy versus Vitrectomy for the Management of Primary Rhegmatogenous Retinal Detachment Outcomes Randomized Trial (PIVOT) | Irrelevant outcomes |
| 2 | Ahmet Burak Bilgin | 2019 | Pars plana vitrectomy with or without intraoperative 360° peripheral endolaser for rhegmatogenous retinal detachment treatment | Irrelevant interventions |
| 3 | Wei Wei Lee | 2022 | Outer Retinal Folds after Pars Plana Vitrectomy vs. Pneumatic Retinopexy for Retinal Detachment Repair: Post hoc analysis from PIVOT | Irrelevant interventions |
| 4 | Vinod Kumar | 2022 | PNEUMATIC VITREOLYSIS VERSUS PARS PLANA VITRECTOMY IN FOCAL SYMPTOMATIC VITREOMACULAR TRACTION SYNDROME: A Randomized Trial | Irrelevant outcomes |
| 5 | Rajeev H Muni | 2021 | Postoperative Photoreceptor Integrity Following Pneumatic Retinopexy vs Pars Plana Vitrectomy for Retinal Detachment Repair: A Post Hoc Optical Coherence Tomography Analysis From the Pneumatic Retinopexy Versus Vitrectomy for the Management of Primary Rhegmatogenous Retinal Detachment Outcomes Randomized Trial | Irrelevant outcomes |
| 6 | Arnd Heiligenhaus | 2003 | Combined phacoemulsification and pars plana vitrectomy: clear corneal versus scleral incisions: prospective randomized multicenter study. | Non-RRD |
| 7 | Ahmad Mirshahi | 2022 | Pars plana vitrectomy combined with phacoemulsifcation versus pars plana vitrectomy only for treatment of phakic rhegmatogenous retinal detachment: a systematic review and meta‑analysis | Systematic review |
| 8 | Chuandi Zhou | 2015 | AIR VERSUS GAS TAMPONADE IN RHEGMATOGENOUS RETINAL DETACHMENT WITH INFERIOR BREAKS AFTER 23-GAUGE PARS PLANA VITRECTOMY: A Prospective, Randomized Comparative Interventional Study | Irrelevant interventions |
| 9 | B Wimpissinger | 2008 | 23-Gauge versus 20-gauge system for pars plana vitrectomy: a prospective randomised clinical trial | Irrelevant interventions |
| 10 | H Ahmadieh | 1999 | Primary capsulectomy, anterior vitrectomy, lensectomy, and posterior chamber lens implantation in children: limbal versus pars plana | Non-RRD |
| 11 | Lukas Kellner | 2003 | 25-gauge vs 20-gauge system for pars plana vitrectomy: a prospective randomised clinical trial | Irrelevant interventions |
| 12 | Daniela Süsskind | 2016 | Primary vitrectomy for rhegmatogenous retinal detachment in pseudophakic eyes: 20-gauge versus 25-gauge vitrectomy | Irrelevant interventions |
| 13 | H Heimann | 2001 | Scleral buckling versus primary vitrectomy in rhegmatogenous retinal detachment (SPR Study): design issues and implications. SPR Study report no. 1 | Irrelevant outcomes |
| 14 | Jonathan Bonnar | 2023 | Scleral Buckle, Vitrectomy, or Combined Surgery for Inferior Break Retinal Detachment: Systematic Review and Meta-Analysis | Systematic review |
| 15 | Vinod Kumar | 2022 | PNEUMATIC VITREOLYSIS VERSUS PARS PLANA VITRECTOMY IN FOCAL SYMPTOMATIC VITREOMACULAR TRACTION SYNDROME: A Randomized Trial | Non-RRD |
| 16 | Mario R Romano | 2017 | TWENTY-SEVEN-GAUGE VERSUS 25-GAUGE VITRECTOMY FOR PRIMARY RHEGMATOGENOUS RETINAL DETACHMENT | Irrelevant interventions |
| 17 | Morteza Mahdizadeh | 2008 | Anatomical retinal reattachment after scleral buckling with and without retinopexy: a pilot study | Irrelevant interventions |
| 18 | Ahmed Saad Albalkini | 2022 | CHANDELIER-ASSISTED VERSUS STANDARD SCLERAL BUCKLING FOR PRIMARY RHEGMATOGENOUS RETINAL DETACHMENT: A Randomized Clinical Study | Irrelevant interventions |
| 19 | Nicolas Feltgen | 2013 | Scleral buckling versus primary vitrectomy in rhegmatogenous retinal detachment study (SPR study): Risk assessment of anatomical outcome. SPR study report no. 7 | Irrelevant outcomes |
| 20 | Amirhossein Roshanshad | 2023 | Pneumatic Retinopexy Versus Pars Plana Vitrectomy for the Management of Retinal Detachment:A Systematic Review and Meta-Analysis | Systematic review |
| 21 | Nicole Heussen | 2011 | Scleral buckling versus primary vitrectomy in rhegmatogenous retinal detachment study (SPR Study): predictive factors for functional outcome. Study report no. 6 | Irrelevant outcomes |
| 22 | Sabine Baumgarten | 2018 | Vitrectomy with and without encircling band for pseudophakic retinal detachment with inferior breaks: VIPER Study Report No. 3 | Irrelevant outcomes |
| 23 | Xi Ling Yu | 2014 | Releasable encircling band for primary rhegmatogenous retinal detachment | Irrelevant interventions |
| 24 | Francesco Morescalchi | 2021 | Pneumatic retinopexy preceded by drainage of subretinal fluid for the treatment of severe bullous retinal detachment | Irrelevant interventions |
| 25 | Prem A.H. Nichani | 2022 | Scleral Buckling Alone or in Combination with Pars Plana Vitrectomy for Rhegmatogenous Retinal Detachment Repair: A Meta-Analysis of 7,212 Eyes | Systematic review |
| 26 | Suthasinee Sinawat | 2010 | Air vs perfluoropropane gas in pneumatic retinopexy: a randomized noninferiority trial | Irrelevant interventions |
| 27 | P E Tornambe | 1991 | Pneumatic retinopexy. A two-year follow-up study of the multicenter clinical trial comparing pneumatic retinopexy with scleral buckling | Irrelevant interventions |
| 28 | Ates Yanyali | 2007 | Steamroller versus basic technique in pneumatic retinopexy for primary rhegmatogenous retinal detachment | Irrelevant interventions |
| 29 | Pasquale Loiudice | 2021 | Localized versus 360° intraoperative laser retinopexy in cases of rhegmatogenous retinal detachment with mild-to-moderate grade proliferative vitreoretinopathy | Irrelevant interventions |
| 30 | Bingqian Liu | 2020 | Comparison of macular buckling and vitrectomy for the treatment of macular schisis and associated macular detachment in high myopia: a randomized clinical trial | Non-RRD |
| 31 | Asfandyar Asghar | 2011 | Outcome of pars plana vitrectomy with silicon oil versus scleral buckling in primary retinal reattachment surgery | Irrelevant interventions |
| 32 | B Mazinani | 2016 | Vitrectomy with or without encircling band for pseudophakic retinal detachment: a multi-centre, three-arm, randomised clinical trial. VIPER Study Report No. 1—design and enrolment | Irrelevant interventions |
| 33 | M Hammer | 1997 | Complex retinal detachment treated with silicone oil or sulfur hexafluoride gas: a randomized clinical trial | Irrelevant interventions |
| 34 | Amir Sternfeld | 2018 | Effect of Subretinal Fluid Drainage Using Perfluorocarbon During Retinal Detachment Repair on Postoperative Metamorphopsia | Irrelevant interventions |
| 35 | Rajeev H Muni | 2020 | Vision-Related Functioning in Patients Undergoing Pneumatic Retinopexy vs Vitrectomy for Primary Rhegmatogenous Retinal Detachment: A Post Hoc Exploratory Analysis of the PIVOT Randomized Clinical Trial | Irrelevant outcomes |
